# Origin and Evolution of the Sodium -Pumping NADH: Ubiquinone Oxidoreductase

**DOI:** 10.1371/journal.pone.0096696

**Published:** 2014-05-08

**Authors:** Adrian Reyes-Prieto, Blanca Barquera, Oscar Juárez

**Affiliations:** 1 Canadian Institute for Advanced Research and Biology Department, University of New Brunswick, Fredericton, NB, Canada; 2 Biology Department, Rensselaer Polytechnic Institute, Troy, New York, United States of America; University of Lausanne, Switzerland

## Abstract

The sodium -pumping NADH: ubiquinone oxidoreductase (Na^+^-NQR) is the main ion pump and the primary entry site for electrons into the respiratory chain of many different types of pathogenic bacteria. This enzymatic complex creates a transmembrane gradient of sodium that is used by the cell to sustain ionic homeostasis, nutrient transport, ATP synthesis, flagellum rotation and other essential processes. Comparative genomics data demonstrate that the *nqr* operon, which encodes all Na^+^-NQR subunits, is found in a large variety of bacterial lineages with different habitats and metabolic strategies. Here we studied the distribution, origin and evolution of this enzymatic complex. The molecular phylogenetic analyses and the organizations of the *nqr* operon indicate that Na^+^-NQR evolved within the Chlorobi/Bacteroidetes group, after the duplication and subsequent neofunctionalization of the operon that encodes the homolog RNF complex. Subsequently, the *nqr* operon dispersed through multiple horizontal transfer events to other bacterial lineages such as Chlamydiae, Planctomyces and α, β, γ and δ -proteobacteria. Considering the biochemical properties of the Na^+^-NQR complex and its physiological role in different bacteria, we propose a detailed scenario to explain the molecular mechanisms that gave rise to its novel redox- dependent sodium -pumping activity. Our model postulates that the evolution of the Na^+^-NQR complex involved a functional divergence from its RNF homolog, following the duplication of the *rnf* operon, the loss of the *rnfB* gene and the recruitment of the reductase subunit of an aromatic monooxygenase.

## Introduction

**Figure 1:**
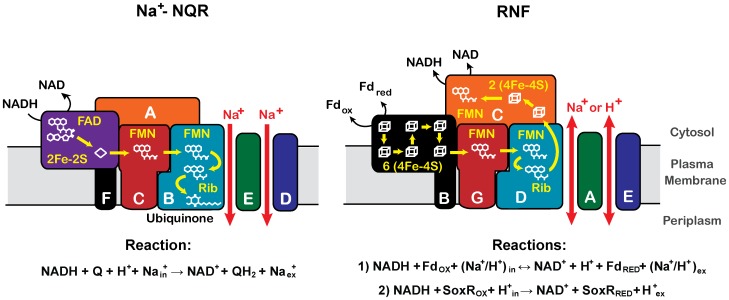
Physiological activities, topology predictions, subunit and cofactor composition and electron transfer pathways of Na^+^-NQR and RNF. Na^+^-NQR catalyzes the transfer of electrons from NADH to ubiquinone, which under physiological conditions is irreversible, and uses the free energy released by the redox reaction to pump sodium [1]–[3]. On the other hand, in most bacterial phyla RNF catalyzes the transfer of electrons between NADH and ferredoxin, which can be reversible depending on the metabolic adaptations of the bacteria [24], [25]. However, the evidence indicates that RNF can also catalyze the reduction the transcriptional regulatory protein SoxR [63], which controls the expression of proteins involved in reactive oxygen species handling. In contrast with Na^+^-NQR, which is specific for sodium [11], the RNF complex might use sodium and protons [25]. The pathway for electron transport in Na^+^-NQR is shown in the yellow lines [1], as well as the proposed pathway for RNF [62]. Ubiquinone is a hydrophobic molecule located in the plasma membrane of bacteria. It has been demonstrated previously that the reduction of this compound occurs in the cytosolic face of the membrane [2], but for illustrative purposes it is shown on the bottom of the figure.

The sodium -dependent NADH: ubiquinone oxidoreductase (Na^+^-NQR) is a bacterial respiratory complex that catalyzes the transfer of electrons from NADH to ubiquinone, and thus it is the gateway by which the redox equivalents, produced by the primary and intermediary metabolism, enter into the respiratory chain [1]–[3] (Figure 1). Na^+^-NQR is not only the first enzyme in the respiratory chain but also one of the main ion transporters in the cell, since the energy released by the redox activity is coupled to the pumping of sodium to the periplasmic space. Thus, Na^+^-NQR coordinates the operation of the entire metabolism with the mechanisms used for osmotic regulation. Na^+^-NQR is commonly associated with the aerobic respiratory metabolism of pathogenic bacteria, such as *Vibrio cholerae* [4], *Vibrio harveyi* [5], *Klebsiella pneumoniae* [6], *Haemophilus influenzae* [7], among others, in which it plays essential roles in different homeostatic functions [8], [9]. Although, a comprehensive study of the role that this enzyme plays in pathogens has not been carried out, the evidence indicates that Na^+^-NQR has an important role in infection, regulating the production of virulence factors [10], and the survival of the cells in the internal host environment. The enormous amount of information that has been obtained with the sequencing of prokaryotic genomes has shown that the genes encoding the subunits of this respiratory enzyme are also found in the genomes of diverse types of bacteria, including aerobic, microaerophilic, obligate anaerobes, photosynthetic, chemolithotrophic, halophilic, alkalophilic and predatory microorganisms (Table S1).

Na^+^-NQR is a sodium -specific ionic pump [11], composed of six subunits (NqrA-F) (Figure 1, Table 1) encoded in the *nqr* operon, that contains five redox cofactors: FAD and a 2Fe-2S center, located in the NqrF subunit, two covalently -bound FMN molecules, located in the NqrB and NqrC subunits, and riboflavin (Table 1) [1]. The binding sites for NADH and ubiquinone are located in the NqrF [12], [13] and NqrB subunits [14], respectively, and although there are some indications suggesting that the riboflavin binding site might be located in NqrB subunit [15], the exact localization of this cofactor remains elusive. On the other hand, the subunits NqrB, NqrD and NqrE contain several transmembrane helices [16], with a large number of acid residues that could be involved in sodium binding and transport [17]–[19]. In contrast with other subunits, NqrA is cytosolic, does not contain either transmembrane segments or cofactor binding sites [1], [20], and although there is controversial evidence about its function [14], [21], [22], it has been considered to fulfill structural roles, since its elimination prevents the association of the complex [15]. Na^+^-NQR is evolutionary related to a membrane -bound NADH: ferredoxin dehydrogenase that was originally described in *Rhodobacter capsulatus*, in which it might have roles in Nitrogen fixation, and thus it is called RNF (Rhodobacter Nitrogen Fixing) [23], [24]. Although in most cases RNF catalyzes the reversible electron transfer between NADH and ferredoxin [25]–[30], coupled to the sodium or proton transport, its function can vary enormously depending on the metabolic adaptations of the bacteria in which it is found (see below). The RNF complex is also composed of six subunits, encoded in the *rnf* operon, most of which are homologous to the subunits of Na^+^-NQR (Figure 1, Table 1, Table S2) [20], [24], [25].

**Table 1:** Structural and catalytic motifs of Na^+^-NQR and RNF subunits.

Subunit/	TH	Cofactors	Substrate binding sites	Function
homologue				
NqrA/RnfC		None (NqrA)/	None (NqrA)/	Structural? (NqrA)/
		2(4Fe-4S) and FMN (RnfC)	NAD^+^ (RnfC)	NAD^+^ reduction (RnfC)
NqrB/RnfD	9/8	Covalently -bound FMN and Riboflavin (?)	Ubiquinone (NqrB) and Sodium	Ubiquinone reduction (NqrB) and Sodium transport
NqrC/RnfG	2	Covalently -bound FMN		
NqrD/RnfE	6		Sodium	Sodium transport
NqrE/RnfA	6		Sodium	Sodium transport
NqrF	1	FAD and 2Fe-2S	NADH	NADH oxidation
RnfB	1–2	6(4Fe-4S) and 2Fe-2S?	Ferredoxin	Ferredoxin oxidation

The cofactor, subunit composition and topology of Na^+^-NQR and RNF are reported previously [1], [25]. TH; Transmembrane Helices.

Na^+^-NQR has many characteristics that are rather uncommon among respiratory enzymes. For instance, it is the only enzyme reported that can use riboflavin (vitamin B_2_) directly as a redox cofactor [31]. Until recently, it was generally accepted that riboflavin acts exclusively as precursor in the biosynthesis of FAD and FMN. Hence, the role of riboflavin as a cofactor in Na^+^-NQR is completely novel. Moreover, Na^+^-NQR produces several flavin radicals during the catalytic cycle, and surprisingly also contains stable flavin semiquinone radical species in different inactive forms of the enzyme [32], an extremely unusual characteristic. Indeed, riboflavin is actually found as a neutral semiquinone radical [31], [32], which is stable in the presence of oxygen and other strong oxidizing agents, indicating that Na^+^-NQR presents a mechanism, like no other reported so far, to stabilize the unpaired electron of the flavin. Furthermore, the binding of the two FMN molecules to NqrB and NqrC subunits involves a phosphoester bond [33], which is also very unusual and can only be found in NqrB and NqrC, their RNF homologues (Table 1) [34] and in the regulatory proteins of N_2_O reductase expression, NOsR and NirI [35]. Although the catalytic mechanisms of the respiratory and photosynthetic enzymes are diverse, the ion pumping strategies of these enzymes can be grouped into two categories: redox loop mechanisms, such as the Q -cycle (i.e., *bc*_1_ complex and photosystem II) [36], [37] and the localized or “direct” coupling (used by cytochrome *c* oxidase and complex I) [38], [39]. Under these two mechanisms the reduction of a molecule or cofactor in a hydrophobic environment is involved in the electroneutral ion capture, and its release on the other side of the membrane, upon oxidation. According to our data, Na^+^-NQR does not follow these ion -pumping strategies. Instead, ion translocation by this complex involves a series of conformational states that allow the capture of sodium in the internal side of the membrane and its release on the opposite side [1], [40]. Given all of these characteristics, it is evident that Na^+^-NQR has evolved separately from other redox enzymes and ion transporters, which has led to the arising of novel enzymatic strategies. In order to gain insights into the evolution and phylogenetic history of Na^+^-NQR, we analyzed the distribution and organization of the *nqr* operon in diverse pathogenic and non -pathogenic bacteria. Our results allow us to hypothesize and discuss a series of molecular events that may have been important in the origin, evolution and spreading of Na^+^-NQR, as well as to postulate some ecological factors that could have been important in its spreading and evolution. The data indicate that Na^+^-NQR appeared originally in the Chlorobi/Bacteroidetes (C/B) phyla, after a duplication of the *rnf* operon. The subsequent loss on the *rnfB* gene prompted significant changes in the mechanism of the enzyme, which together with the recruitment of the reductase subunit of the aromatic (phenol, toluene and bencene) monooxygenases (AMOr protein) underlie the origin of Na^+^-NQR. The current distribution of this complex in diverse bacterial lineages can be explained by at least two ancestral independent horizontal gene transfer (HGT) events, to proteobacteria, and Chlamydiae and Planctomyces, and at least two posterior HGT events, from Chlorobi to δ-proteobacteria and from Pasteurellales (γ-proteobacteria) to Neisseriales (β-proteobacteria).

## Materials and Methods

### Homolog Identification, Multiple Sequence Alignment and Phylogenetic Analyses

Using the amino acid sequences of the Na^+^-NQR subunits of *Vibrio cholerae* (O1 El Tor strain N16961) orthologous sequences were identified in the RefSeq GenBank database. After an exhaustive similarity search (blastp; cutoff E-value ≤1e-15), protein sequences were retrieved, covering all major bacterial groups where Na^+^-NQR subunits were identified (Table S1). The *nqr* operon was not found in the bacterial groups Acidobacteria, Actinobacteria, ε-proteobacteria, Aquificae, Chloroflexi, Cyanobacteria, Deinococcus/Thermus and Thermotogae, neither in Archaea or Eukaryotes (Table 2). Our final protein data set comprised sequences of the six Na^+^-NQR subunits, from 90 bacterial taxa (Table S1). The six protein sets were aligned with MAFFT v7 [41], using the FFT-NS-I method, with the BLOSUM 62 substitution matrix. After visual inspection to discard incomplete protein models, fused or chimeric sequences, the raw multiple alignments were processed with Gblocks v0.91b [42], to discard poorly aligned positions and ambiguous regions. In all cases the alignments obtained after Gblocks processing contained all the catalytically and structurally relevant sections of the six subunits. Interestingly, the alignment of the NqrA subunits showed that the sequence of this subunit is highly variable. This variability is possibly related to relaxed functional constraints, given the lack of catalytic function of NqrA, reflected in a relatively high amino acid substitution rate. To test the hypothesis of homology (i.e., common origin) of the Na^+^-NQR and RNF subunits we used the global alignment software Needdle (EMBOSS) to estimate the pairwise percentage of sequence identity (PID) between Na^+^-NQR and RNF putative homolog pairs, and NqrF with the AMOr protein. We carried out two independent intraspecific sequence comparisons using the Na^+^-NQR and RNF proteins from both *Chlorobium phaeobacteroides* (Chlorobi) and *Anaerophaga thermohalophila* (Bacteroidetes), respectively. A single randomly selected AMOr sequence (*Cupriavidus metallidurans* CH34) was aligned independently against the *Chlorobium* and *Anaerophaga* nqrF subunit.

**Table 2:** Distribution of Na^+^-NQR and RNF in Archaea and Bacteria.

Domain	Group	RNF						Na^+^-NQR					
		C	D	G	E	A	B	A	B	C	D	E	F
**Bacteria**	Acidobacteria												
	Actinobacteria	▪	▪	▪	▪	▪	▪						
	α -proteobacteria	▪	▪	▪	▪	▪	▪	▪	▪	▪	▪	▪	▪
	β-proteobacteria	▪	▪	▪	▪	▪	▪	▪	▪	▪	▪	▪	▪
	γ-proteobacteria	▪	▪	▪	▪	▪	▪	▪	▪	▪	▪	▪	▪
	δ-proteobacteria	▪	▪	▪	▪	▪	▪	▪	▪	▪	▪	▪	▪
	ε -proteobacteria												
	Aquificae												
	Chlamy/Verrua							▪	▪	▪	▪	▪	▪
	Planctomycetes							▪	▪	▪	▪	▪	▪
	Chlorobi	▪	▪	▪	▪	▪	▪	▪	▪	▪	▪	▪	▪
	Bacteroidetes	▪	▪	▪	▪	▪	▪	▪	▪	▪	▪	▪	▪
	Chloroflexi												
	Cyanobacteria												
	Deinococcus/Thermus												
	Firmicutes	▪	▪	▪	▪	▪	▪						
	Fusobacteria	▪	▪	▪	▪	▪	▪						
	Spirochaetes	▪	▪	▪	▪	▪	▪						
	Tenericutes	▪	▪	▪	▪	▪	▪						
	Thermotogae	▪	▪	▪	▪	▪	▪						
**Archaea**	Methanosarcinales	▪	▪	▪	▪	▪	▪						

Please note that although the *nqr* and *rnf* operons were found in these phyla, there is a large variability among the bacterial orders and some of them might not contain any of the two complexes. Moreover, the presence of the genes might not indicate that the complexes are functional and further biochemical characterizations are necessary to clarify these and other aspects.

The intraspecific sequence similarity surveys revealed normalized PIDs of at least 30% in four out of six tested homolog pairs (see PID2-PID4 values in Table S2). According with typically used PID thresholds (30%) [43]–[45], the identity values between RnfE/NqrD, RnfA/NqrE, RnfD/NqrB and AMOr/NqrF (Table S2) indicate that these pairs are homologous, supporting previous works [2], [25]. The PID values in the case of the RnfC/NqrA and RnfG/NqrC pairs were between 22 and 33% depending on the normalization strategy. Even though the PID in these last two Na^+^-NQR/RNF pairs are in the “twilight zone” [46] to clearly define families of homologous proteins, the hypothesis of common origin is substantially reinforced by structural similarities and shared protein domains observed between these protein duos, in particular in the case of NqrC/RnfG, which present a similar topology and conserved motifs for the interactions with the covalently -bound FMN cofactor (Table 1) [25], [34]. In the case of NqrA/RnfC, the evolutionary relationship of these subunits has been difficult to establish, due to the borderline PID values (22–27%). To offer further evidence supporting the homology of these subunits, secondary structure prediction analysis were performed and illustrated together with the pairwise sequence alignment of the NqrA and RnfC subunits of *A. thermohalophila* (Fig. S1). Both subunits show strong similarities in the amino terminus, containing a barrel sandwich hybrid domain and remnants of the NADH, FMN and Fe-S binding domains. The secondary structure predictions show that these two proteins have a very similar (>80%) structure (and probably a similar folding pattern), which together with their relative positions in the operon (see below), and the PID values, support the homology hypothesis.

To gain additional insights into the evolution of the Na^+^-NQR complex, we generated multiple alignments of each of the Na^+^-NQR subunits, together with the homologous proteins that constitute the RNF complex (see Table 1 for Na^+^-NQR/RNF homolog-pair details) [24], [25]. NqrF is the only Na^+^-NQR subunit with no homologous counterpart in the RNF complex. Our PSI blast similarity analysis showed that the AMOr subunit is the closest homolog to NqrF, sharing the FAD, NADH and 2Fe-2center binding sites [47]. Thereafter, we were able to generate multiple alignments of the homolog pairs: NqrB/RnfD, NqrD/RnfF, NqrE/RnfA and NqrF/AMOr. The high divergence in the amino acid sequence of the homolog pairs NqrA/RnfC and NqrC/RnfG precluded the estimation of reliable multiple alignments for phylogenetic analysis.

The best amino acid substitution model for each Na^+^-NQR subunit alignment was selected according to ProtTest. The Le and Gascuel (LG) substitution model [48], considering a gamma distribution of substitution rates (+G) and a proportion of invariable sites (+I), was selected in all cases. ML trees using the LG+G+I substitution model were estimated with RAxML HPC-MPI 7.2.8 [49] with 500 bootstrap pseudoreplicates. Bayesian posterior probabilities were calculated with MrBayes 3.2.1 [50], considering the WAG+G+I substitution model (LG model is not implemented in MrBayes) running a Metropolis-coupled Markov Chain Monte Carlo (MC3) for 2 million generations. Two independent MC3 runs were performed simultaneously, with 1 “cold” and 3 “heated” chains starting from different random trees. Chain convergence was evaluated every 250,000 generations, until the average standard deviation of split frequencies dropped below 0.01. The pool of trees was sampled every 100th generation. Final posterior probabilities were estimated after discarding the trees of the first 500,000 generations. Visual inspection of trees was carried out with Archaeopteryx 0.997 beta version [51].

## Results and Discussion

### Distribution and Metabolic Roles of Na^+^-NQR

The RNF and Na^+^-NQR complexes are among the few respiratory enzymes that couple their redox activities with the ability to create an electrochemical gradient of sodium (Figure 1) [1], [25]. These two enzymes share numerous characteristics that include a similar cofactor composition, sodium translocation mechanisms and structural motifs (Table 1) [25], [27], [28]. Nonetheless, one of the main differences between RNF and Na^+^-NQR is the mechanism used to incorporate electrons into the complex. In the case of Na^+^-NQR, electrons are introduced into the transport chain through NqrF [12], [52], and in the case of RNF, it has been proposed that the redox equivalents are introduced from ferredoxin, using the RnfB subunit [25]. NqrF and RnfB subunits are complex-specific and have no homologous counterpart in RNF or Na^+^-NQR.

The *rnf* operon is present in numerous bacterial lineages and can also be found in the Methanosarcinales order from the domain Archaea (Table 2). In contrast, the *nqr* operon is exclusively found in bacteria, and it is absent in both eukaryotes and archaea. Na^+^-NQR has been extensively studied in marine/opportunistic pathogens, especially in γ -proteobacteria, such as *V. cholerae* [4], [9], in which it forms part of the aerobic respiratory chain. However, our results now show that the *nqr* operon is found in bacteria with diverse metabolic capabilities and life-styles, including photosynthetic, chemolithotrophic and anaerobic bacteria (Table S1). In spite that the Na^+^-NQR complex is found in distantly related bacterial groups, such as α, β, γ and δ -proteobacteria, Planctomyces, Chlamydiae, Chlorobi and Bacteroidetes, it is apparently absent in many major lineages (Table 2). Na^+^-NQR patchy distribution suggests that this enzymatic complex was not present in the most recent common bacterial ancestor. Overall, the *rnf* operon presents a wider distribution among major bacterial lineages than Na^+^-NQR. Interestingly, in some bacterial lineages, such as Chlorobi/Bacteroidetes and most proteobacteria, both homologous complexes coexist (Table 2).

Na^+^-NQR -containing bacteria can be classified according to their habitat into marine/halophilic and endo/pathogenic bacteria. Pathogenic bacteria harboring Na^+^-NQR include β and γ -proteobacteria (Enterobacteriales, Vibrionalles, Pasteurellales, Aeromonadales, Pseudomonadales, Neisserales), Bacteroidetes and Chlamydiae, the latter are obligate intracellular parasites [8]. Most free -living organisms (as well as mitochondria and plastids) produce a transmembrane gradient of protons to convert the free energy released by different chemical reactions, into electrochemical energy that can be used to sustain a variety of processes, such as ATP synthesis. In contrast, pathogenic bacteria might not be able to use protons as energy currency, mostly because the extracellular pH is more alkaline (plasma pH = 7.2–7.4), compared to the internal pH of the cell (pH 6.0–6.5) [53], [54], and thus they are actually facing an “inverted” proton gradient [55]. Instead of using protons, pathogenic bacteria would produce a gradient of sodium, which is the most common cation in the plasma. It has been proposed that the sodium gradient is essential for survival of bacterial parasites, because it is used to support a large variety of processes, including pH regulation, ATP synthesis, cell motility, uptake of nutrients, toxin extrusion, as well as the efflux of drugs in antibiotic- resistant strains [8], [55]. Indeed, the evidence indicates that Na^+^-NQR plays an essential role in the physiology of many pathogens. In fact, the expression of Na^+^-NQR increases greatly during the infection of the small intestine by *V. cholerae* [56], and random insertion mutagenesis has demonstrated that the inactivation of *nqr* operon reduces drastically the infectivity of these cells [57]. Moreover, Na^+^-NQR activity regulates the expression of the *V. cholerae* virulence factors, such as the cholera toxin (CTX) and the major subunit of the toxin co-regulated pillus (TcpA), through the modulation of the ToxR, TcpP, and ToxT regulatory cascade [10].

Marine/halophilic bacteria containing Na^+^-NQR include Plactomycetes, Chlorobi, as well as α, δ, free- living β and “early diverging” γ-proteobacteria. All these types of bacteria have different metabolic strategies, including photoautotrophic or chemolitotrophic anaerobes, chemorganothrophs, as well as strict aerobic bacteria. In these organisms, Na^+^-NQR pumping activity could be used both to generate a gradient of sodium, coupled to the activity of the oxidative phosphorylation, and to maintain the intracellular ionic composition. Indeed, in the extreme haloalkalophile *Desulfurivibrio alkaliphilus* (which is a sulfate reducing bacteria -see below- found in soda lakes [58]), the activity of Na^+^-NQR should be an essential part of the mechanisms used for ionic homeostasis. Interestingly, Na^+^-NQR is also found in the predatory δ -proteobacteria *Bacteriovorax marinus*, in which its activity might be employed to form an electrochemical gradient, driving flagellum rotation. Ion -driven cell motility is essential for predatory bacteria, since is the main strategy to find prey bacteria [59].

The *nqr* operon is also found in sulfate reducing (SR) bacteria, such as δ -proteobacteria, especially in marine species. In these bacteria Na^+^-NQR might participate in osmotic protection, but may have other roles in the metabolism, especially in menaquinone reduction. Menaquinol is the main electron donor in the first stage of sulfate -based respiration. The Qmo complex catalyzes the menaquinol -mediated reduction of the activated sulfate (APS), which is converted to sulfite. Further reduction of sulfite to sulfuric acid requires electrons provided by periplasmic hydrogenases [60]. The full reduction of sulfate to sulfur requires a large amount of redox equivalents (8 electron pairs) and 2ATP molecules, thus it has been postulated that several respiratory enzymes such as RNF [61] or Na^+^-NQR could have an important role in this process.

### Horizontal Gene Transfer in the Evolution of *nqr* Operon

One of the distinguishing aspects of RNF is that its function can vary enormously, depending on the metabolic adaptations of the bacteria in which is found. For instance, in *Acetobacterium woodii* (anaerobic acetogenic), RNF catalyzes the reduction of NAD (−320 mV), using the redox equivalents of Ferredoxin (−500 mV) [25], [62]. This can be considered as the “forward reaction”, since the electron transfer is downhill, and the energy released is used by the enzyme to pump sodium (Figure 1). In *Rhodobacter capsulatus* (purple non -sulfur photosynthetic), and probably in other nitrogen fixing bacteria, RNF uses the electrochemical gradient to drive the backwards reaction, reducing ferredoxin, using NADH as electron donor [27]. On the other hand, in *E. coli* RNF transfers the electrons from NADH, to the low molecular weight SoxR protein, which is a transcription factor that controls the expression of proteins involved in oxidative stress protection [63]. In addition, indirect evidence suggest that in some cases RNF could function as a proton pump [64]. Thus, it seems that the ability of RNF to sustain major catalytic innovations, conserving the basic structural and catalytic mechanisms, may have been a key factor in the evolution of Na^+^-NQR.

The fact that the *nqr* operon is found only in certain bacterial lineages, while the *rnf* operon is widely distributed (Table 2), suggests that RNF might have been the enzymatic ancestor of Na^+^-NQR. The common ancestry of 5/6 of the Na^+^-NQR and RNF subunits posits a unique scenario to investigate the origin and evolution of these enzymatic complexes from a common ancestral gene cluster. In order to investigate the origin of the Na^+^-NQR components, we estimated ML trees from multiple alignments generated for each Na^+^-NQR/RNF homolog pairs (NqrB/RnfD, NqrD/RnfF, NqrE/RnfA), defining the RNF clades as outgroups (see Table 1 and Methods section for Na^+^-NQR/RNF homolog-pair details). The NqrF and RnfB subunits are complex-specific and they have no homologous counterpart in RNF or Na^+^-NQR, respectively. Thereafter, a multiple alignment NqrF/AMOr was generated as well. Each of the four rooted ML trees (Figs. S2–S5) consistently depict C/B and some SR δ -proteobacteria (of the family Desulfobacteraceae) as the earliest diverging sequences within the Na^+^-NQR subunits. Given the consistent early branching position of the C/B and the “anomalous” position of the SR Desulfobacteraceae nested within them, we hypothesize that the Na^+^-NQR evolved first within the Chlorobi/Bacteroidetes group, after an ancestral duplication and posterior neofunctionalization of some elements the *rnf* operon (see discussion below). The phylogenetic analysis suggests that other bacterial lineages acquired the Na^+^-NQR complex via multiple HGT events (see below).

**Figure 2:**
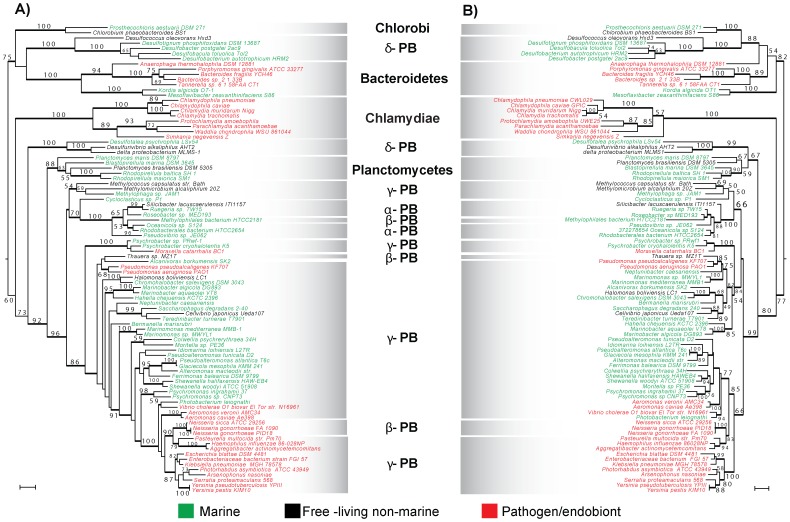
Maximum Likelihood phylogenetic analyses of NqrB (A) and NqrF (B) subunits of the Na^+^-NQR complex. Numbers near nodes indicate RaxML bootstrap branch support values (when ≥50%) and Bayesian posterior probabilities ≥0.95. Branch lengths are proportional to the number of substitutions per site (scale bars corresponds to 0.1 substitutions per site, respectively). Major bacterial groups are highlighted with shaded boxes. Taxa in red characters are endobionts or pathogens. Organisms in green type set have been found in marine habitats and those in black characters are free-living non-marine bacteria.

The ML trees shown in figure 2 were estimated with the sequences of subunits NqrB (Fig. 2A) and NqrF (Fig. 2B), respectively, and rooted in the C/B and SR Desulfobacteraceae node. Overall, the topologies of both trees show important consistencies. The Chlamydiae group branches immediately after the defined rooting node. However this position has to be taken with caution given that the Chlamydiae *nqr* operon is split in three fragments [65], [66] and it is evident the Chlamydiae clade has evolved through a high amino acid replacement rate. The main branch of SR δ -proteobacteria and Planctomyces are found immediately after the Chlamydiae cluster, these two groups are united in a single branch (67% bootstrap support; BS) in the NqrF tree (Fig. 2B), whereas branch separated in NqrB (Fig. 2A). Both ML trees depict a strongly supported clade (92% and 99% BS, respectively) comprising the diverse proteobacterial lineages. Overall, the NqrB (414 residues long in *Vibrio cholerae*) and NqrF (404 residues) ML trees show considerable resolution in different nodes and it is likely that the catalytic roles of NqrB and NqrF have restrained the substitution rate in the catalytic regions, but allow higher variability in non-essential regions, producing a relatively consistent phylogenetic signal. The topologies of the ML trees of the short subunits NqrD (Fig. S6; 210 residues), NqrE (Fig. S7; 198 residues), NqrC (256 residues; Fig. S9) and the longer NqrA (414 residues; Fig. S8) show some important differences with respect the positions of the Chlamydiae long-branch and the “anomalous” SR δ-proteobacteria branches (i.e., C/B are not branching in the same clade with Chlamydiae and/or SR δ-proteobacteria intermingled; Fig. S6 and S7), therefore in these cases rooting in the Chlorobi/Bacteroidetes clade was not attainable. Even though the resolution and branch support values are relatively low in the NqrD and NqrE trees, similar topological patterns within the proteobacterial clades are consistent with the NqrB and NqrF results. Overall the ML tree topologies suggest a common history underlying the evolution of the whole Na^+^-NQR complex.

**Figure 3:**
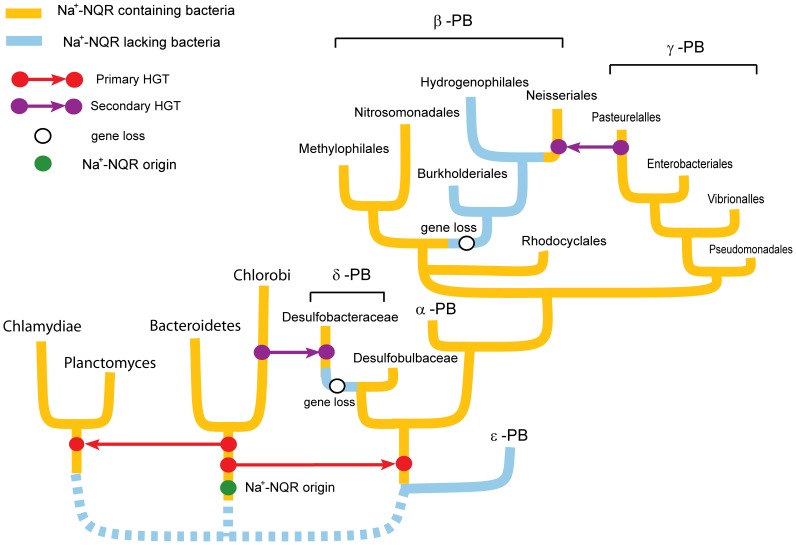
Multiple horizontal gene transfer (HGT) events involved in the dispersal of the *nqr* operon. Our data indicate that Na^+^-NQR appeared in the common ancestor of the Chlorobi and Bacteroidetes groups. The operon was transferred by HGT to Chlamydiae and Planctomyces, and to the ancestor of α, β, γ and δ–proteobacteria, after this lineage split from ε -proteobacteria. The evidence indicates that both Desulfobacteraceae (δ–proteobacteria) and the ancestor of Burkholderales, Hydrogenophilales and Neisseriales (β–proteobacteria), lost the *nqr* operon and was reacquired later. In the case of Desulfobacterales, from Chlorobi, and in the case of Neisseriales, from pathogenic Pasteurelalles (γ–proteobacteria).

We hypothesize that after the emergence of the Na^+^-NQR in C/B, it was subsequently dispersed to other bacterial lineages by, at least, three independent HGT events (Figure 3): to the Chlamydiae and Planctomyces ancestor and in two different occasions to proteobacteria. The results suggest that the common ancestor of α, β, γ and δ -proteobacteria originally recruited Na^+^-NQR via HGT, since it is not found in ε -proteobacteria. Indeed, the topology of the proteobacterial branch resembles the consensus tree of this bacterial lineage, with α, β and δ -proteobacteria in the base of the tree [67]. Also, our trees display a pattern that is similar to γ-proteobacteria phylogeny, with Pasteurelalles and Enterobacteriales in the top branches [68]. It must be pointed out that the minor differences in the proteobacterial clade found in our single-locus trees, in comparison to evolutionary patterns found elsewhere [67], are not unexpected, since those trees were estimated by consensus analysis of dozens of different proteins. The phylogenetic analyses of NqrB (Figs. 2A) and NqrF (Figs. 2B) show that the evolution of Na^+^-NQR has followed a convoluted pattern inside the proteobacteria lineage, particularly in the case of β and δ -proteobacteria, which appear branching in different locations in the trees. Our data demonstrate that “basal” β-proteobacteria, such as Rhodocyclales (*Thauera* sp. MX1T) and other species, including Candidatus *Accumulibacter phosphatis* (not shown), as well as Nitrosomonadales (*Nitrosomonas europaea* ATCC 19718, not shown) and Methylophilales (*Methylophilales bacterium* HTCC2181) contain Na^+^-NQR, which is closely related to α -proteobacteria and “basal” γ -proteobacteria, matching relatively well their phylogenetic history [67]. Additionally, another β-proteobacteria group, comprising exclusively Neisseriales, is found branching with pathogenic γ-proteobacteria, specifically with Pasteurellales (90% and 83% BS in NqrB and NqrF trees, respectively) and is separated from the main β-proteobacteria branch. Interestingly, the sister groups of the Neisseriales, including Hydrogenophilales and Burkholderiales, lack the *nqr* operon, indicating that the common ancestor of these groups lost the genes coding for Na^+^-NQR. Thus, it is possible that Neisseriales, which are pathogenic, reacquired the *nqr* operon from pathogenic γ-proteobacteria (Figure 3).

As mentioned before, a group of SR δ-proteobacteria (Desulfobacteraceae) branch consistently with Chlorobi/Bacteroidetes (Figs. 2A, 2B). This topology does not reflect the evolutionary history of these non-related bacterial major groups, but instead may indicate a more recent HGT event from C/B into Desulfobacteraceae. This transference may have included other genes, not only Na^+^-NQR, as shown by Biegel *et al*, in the evolutionary analysis of RnfD, which shows almost exactly the same topology [25]. Moreover, SR δ-proteobacteria and Chlorobi contain other metabolic pathways that are not common among bacteria, such as the reductive Tricarboxylic acid cycle [69], [70]. Thus, it appears that these two distantly related bacterial lineages have exchanged a significant amount of genetic information. It should be pointed out that several proteobacteria have lost Na^+^-NQR, most conspicuously *Escherichia coli*. This effect probably represents adaptations of different organisms to their environmental conditions. Surprisingly, the *nqr* operon can also found in the closely related enteric bacteria *Escherichia blattae*.

The ML trees of NqrB and NqrF also show that Na^+^-NQR was likely spread to Chlamydiae and Planctomyces, through one or two lateral ancient HGT events. Phylogenetic trees obtained with 16S rRNA have located Chlamydia and Planctomyces as distantly related groups [71]. Furthermore, peptidoglycan, which is a universal component of bacterial cell walls, is absent in the cell walls of both Chlamydiae [72] and Plantomyces [73], [74]. Interestingly, Chlamydiae contains most of the genes involved in peptidoglycan synthesis [65], [75] and the addition of β-lactam antibiotics blocks its normal development [76], but their cell walls do not contain detectable amounts of peptidoglycans. Thus, it is possible that Na^+^-NQR may have been transferred from C/B to the common ancestor of Chlamydiae and Planctomyces, and that Chlamydiae Na^+^-NQR would have evolved faster, including a fragmentation of the *nqr* operon, than the Planctomyces homologs, explaining the Chlamydiae long branch and the separation of both lineages in the trees.

An important ecological aspect that may have contributed to the evolution and spread of Na^+^-NQR to different bacterial lineages could have been a limited iron availability, which is now reflected on the particular cofactor composition of the “modern” Na^+^-NQR complex. One of the most intriguing aspects of this enzyme is the preferential use of flavin molecules compared to iron containing cofactors [1]. While most respiratory enzymes contain a large number of cofactors that contain iron, including complex I that has up to nine Fe-S centers [77], Na^+^-NQR contains only one 2Fe-2S center and four flavin molecules. Iron has been considered one of the key elements involved in the origin of life, probably forming part of the first cofactors in enzymatic reactions [78], [79]. The ability to use minimal amounts of iron could represent an early adaptation of primordial anaerobic cells to the rising levels of oxygen. While ferrous iron is “life -compatible” and easy accessible under anaerobic conditions, due to its high solubility, under aerobic conditions ferric iron has a very low solubility and can react easily with oxygen, producing superoxide that can damage different cell components [78], [80], [81]. On the other hand, in the case of parasitic organisms, where Na^+^-NQR is well represented [8], [55], the ability of operate with minimal amounts of iron would be advantageous, since an important part of the immunity mechanisms of the body include the limitation of the availability of iron or its complete depletion in the infection site [81], [82].

### The Organizations of the *nqr* Operon and the Origin of the Na^+^-NQR Complex

**Figure 4:**
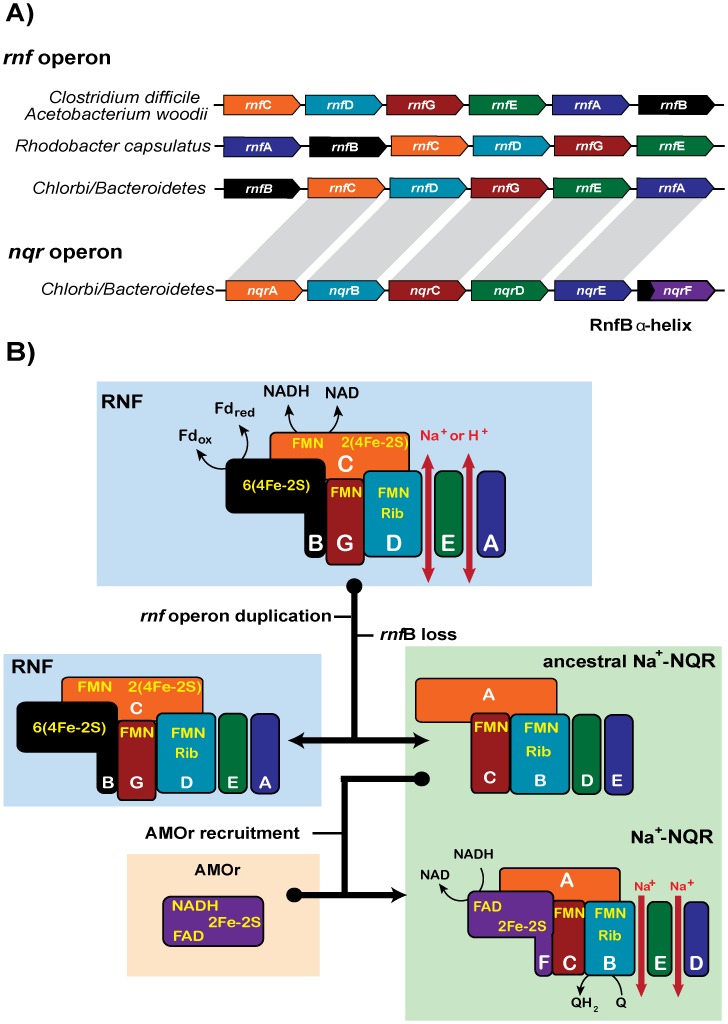
Model of Na^+^-NQR complex origin after the ancestral duplication of the *rnf* operon. **A)** Gene organization of the *rnf* operon in diverse bacterial lineages. The order *rnfBCDGEA* of the Chlorobi/Bacteroidetes *rnf* operon resembles the gene organization *nqrABCDEF* of the homologues genes that constitute the *rnf* operon of most bacteria. **B)** Schematic model for the functional evolution of the Na^+^-NQR complex from a duplicated RNF complex encoded by an *rnf* operon which likely lost the *rnf*B gene. According to our hypothesis subunit NqrF complex evolved from the reductase subunit of aromatic monoxygenase (AMOr) family that recruited the α -helix region of the former RnfB subunit.

The phylogenetic results and the distributions of the *rnf* and *nqr* operons in the major bacterial lineages suggest that Na^+^-NQR appeared from the duplication of the *rnf* operon in the C/B common ancestor. After its putative duplication, the original *rnf* operon kept its function, as sodium -pumping ferredoxin: NADH oxidoreductase, while the paralogue copy lost a key gene (Fig. 4A and 4B). Under our proposal, the gene encoding the subunit RnfB, which is probably involved in the electron uptake from reduced ferredoxin [25], was lost in the *nqr*-predecessor operon after the duplication event, although the core organization of the operon elements (*rnf*CDGEA/*nqr*ABCDE) remained essentially identical. Examination of the *rnf* operon organization in different bacteria offers further evidence to support this hypothesis (Fig. 4A). In organisms such as *R. capsulatus*, and most proteobacteria, the genes in the *rnf* operon are assembled in the order *rnfABCDGE* [83]. The origin of *nqr* operon in these types of bacteria would have not only involved the duplication of the *rnf* operon, but major gene rearrangements, which makes this scenario unlikely. On the other hand, in the C/B lineage, the organization of the *rnf* operon is *rnfBCDGEA*, very similar to the organization found in the typical *nqr* operon [25]. Thus, it is conceivable that the current organization of the *nqr* operon would have appeared first in the C/B *rnf* operon, fact reflected in the conserved order of the homologous genes. There are other bacteria in which the organization of the *rnf* operon could have also led to the evolution of Na^+^-NQR, such as in Firmicutes, including different species of the *Clostridium* genus [84], [85] and *A. woodii* [62], where the organization of the genes is *rnfCDGEB*, but in contrast to the case of C/B, they do not harbor the *nqr* operon (Table 2). It must be pointed out that some authors have proposed that the RNF complex might include other subunits, such as RnfH [83], RnfX, RnfY [86] or endonuclease III [87]. However, these subunits are not found in the *rnf* operons in other bacteria and their role in the function of RNF is still obscure.

**Figure 5:**
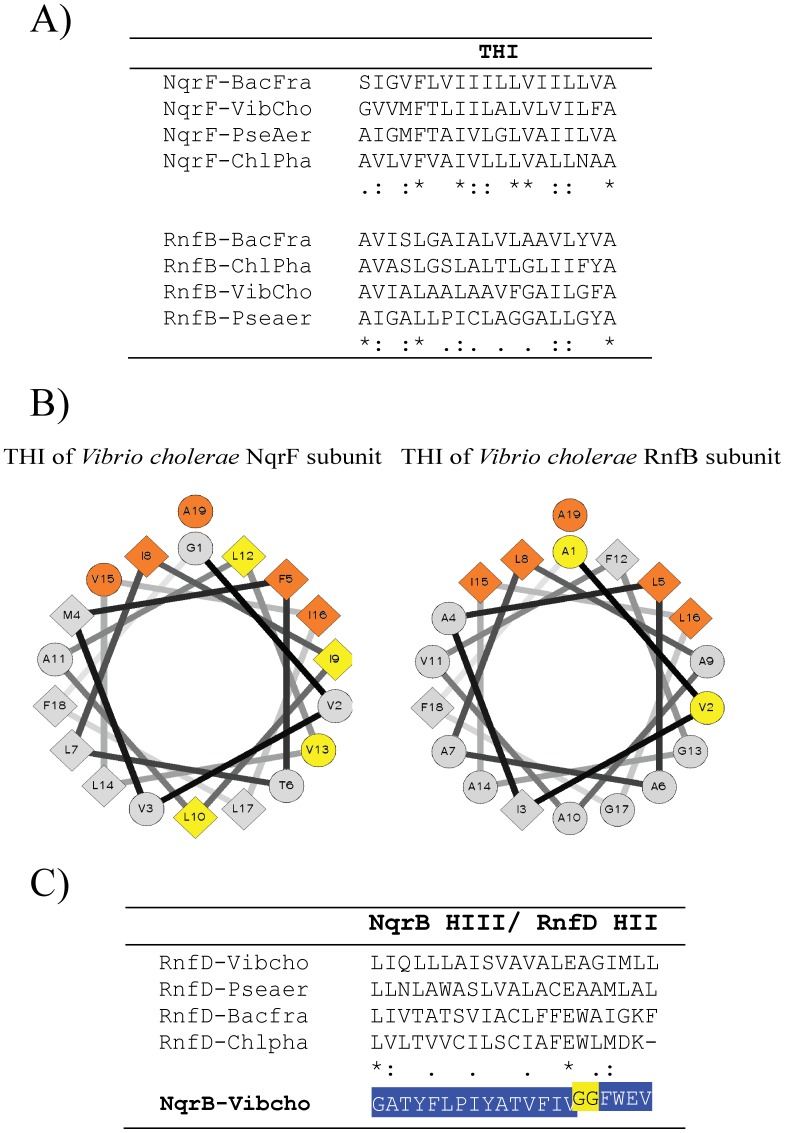
Comparative analysis of Na^+^-NQR and RNF transmembrane helices. A) Multiple sequence alignment of the transmembrane helix I of NqrF and RnfB subunits. B) Helical wheel projection of *Vibrio cholerae* NqrF and RnfB transmembrane helices. Conserved residues within NqrF and RnfB subunits are highlighted in yellow, and residues that are conserved in both NqrF and RnfB are found in orange. C) Multiple sequence alignment of the transmembrane helix II of RnfD subunit compared to the homologous helix III of NqrB subunit. The position of the two conserved glycine residues 140 and 141 in NqrB is highlighted in yellow. In this panel the conserved and semiconserved residues correspond exclusively to the sequences of RnfD. BacFra; *Bacillus fragilis*, VibCho; *Vibrio cholerae*, PseAer; *Pseudomonas aeruginosa*, ChlPha; *Chlorobium phaeobacteroides*.

We propose that the loss of the *rnfB* gene in the duplicated *rnf* operon was the key event that elicited the evolution of Na^+^-NQR, triggering the evolutionary process underlying the change in the function of RNF into Na^+^-NQR, which included the switch in electron acceptor and donor. In Na^+^-NQR, the electrons are accepted by the FAD molecule in subunit NqrF and are transferred according to the pathway shown in Figure 1 [14]. On the other hand, Biegel *et al* [25] postulated that in *A. woodi* RNF, the electrons may enter the enzyme in RnfB subunit, which contains a polyferredoxin domain, with a number of Fe-S centers, and would follow a similar electron pathway as found in Na^+^-NQR (Figure 1). In the scenario depicted in Fig. 4, the loss of RnfB would have rendered the RnfC subunit non -functional, which eventually might have led to the loss of the binding sites for NAD and FMN, as well as the cysteines involved in the binding of the two 4Fe-4S clusters, but nonetheless this subunit retained its structural role, thus giving rise to the NqrA subunit. Indeed, NqrA contains reminiscences of the substrate and cofactor binding sites of RnfC, but the critical residues involved in the binding have been lost (Figure S1). After the RnfB loss, the next step in Na^+^-NQR evolution might have been the recruitment of a “new” electron-accepting module, which is the NADH dehydrogenase module in NqrF. As mentioned before, none of the RNF complex subunits are evolutionary related to NqrF and the closest known homologue is the AMOr subunit. We postulate that a gene encoding an AMOr was recruited by the primordial C/B *nqrABCDE* operon, establishing its function as the reductase module of Na^+^-NQR complex (Fig. 4B). However, a complete integration of this subunit into the complex required the acquisition of a membrane attachment domain, since typical AMOr are hydrophilic proteins with no membrane attachment sites. Although the transmembrane segment is the most variable part of NqrF, it contains the conserved general structure: X_4_-F-X_2_-I-X-(I/V/L/M)-X-L-X_3_-(L/I)-X_3_-(A/S), implying that it is not only an membrane anchor site, but also should be able to interact with the transmembrane segments of other subunits. Remarkably, helix I of the RnfB subunit resembles the transmembrane helix of NqrF, sharing a very similar structure A-(V/I)-X_2_-L-X_2_-(L/I)-X_6_-(V/I/L)-(I/L)-X_2_-(A). The wheel projections of both helices show that positions 5, 8, 15, 16 and 19 have similar residues and are conserved in both transmembrane segments and are concentrated in a relatively small face of the helical cylinder (Figure 5B). Thus, it is likely that the NqrF subunit evolved as the result of the gene fusion of AMOr and the first transmembrane segment of RnfB or that the acquisition of the membrane domain could have followed a convergent evolution pattern, producing a structure similar to the RnfB transmembrane helix that was able to establish the same structural contacts. Under both of these hypotheses, the integration of NqrF into the complex would have been facilitated through the hydrophobic interactions of the transmembrane segment with other subunits.

Moreover, another important part of Na^+^-NQR redox partner switching is the use of ubiquinone as electron acceptor. Clues about this molecular event can be found by examining the topology and sequence of the subunits NqrB and RnfD. Figure 5C shows the sequence alignment of transmembrane helix III of *V. cholerae* NqrB subunit (shown highlighted in blue as reference on the bottom), compared to the homologous region of RnfD (transmembrane helix II) from different bacteria. Helix III in NqrB subunit contains two conserved glycine residues that are involved in the formation of the ubiquinone binding site and whose mutation, even to other small residues, such as alanine or valine, disrupts completely the interaction with ubiquinone [14]. Although these residues might not interact directly with the substrate, they probably create a cavity for ubiquinone. The analysis of the homologous transmembrane segment in RnfD shows that this helix does not contain the two conserved glycine residues. Thus, it is evident that the acquisition of the ubiquinone reductase activity involved the opening of the ubiquinone binding site, through the loss of bulky residues in transmembrane helix III and their substitution by glycine. In addition, our data indicate that Na^+^-NQR originally evolved as a NADH: menaquinone reductase, since ubiquinone (*Em* = +90 mV) is absent in both Chlorobi [88]–[90] and Bacteroidetes [90], [91], and instead they present the low potential analog, menaquinone (*Em* = −80 mV). The ability to use menaquinone might be one of the key elements that determined the spread to other types of bacteria, for instance to δ-proteobacteria and planctomyces, which also contain menaquinone [90]. The ability of Na^+^-NQR to use ubiquinone appeared later on evolution, probably first in aerobic α-proteobacteria and was inherited to β and γ-proteobacteria, all of which contain exclusively ubiquinone [90]. It is important to highlight that the mechanism of sodium translocation by Na^+^-NQR should be essentially the same with the two redox electron acceptors, since the reduction of the quinone molecule is not involved directly in sodium translocation [40]. The role played by riboflavin is another aspect to consider during the transition of the catalytic mechanism of RNF, to become Na^+^-NQR. Our group described that riboflavin is a functional cofactor in Na^+^-NQR, delivering the electrons to the final substrate [31], [52], a very unusual and probably unique case in nature. Little inferences can be done at this point about the evolutionary mechanisms involved in the acquisition of riboflavin, since the location of this cofactor remains elusive. However, W. Buckel’s group found evidence indicating that RNF also contains riboflavin [92]. Thus, it seems that the evolution of Na^+^-NQR did not require the acquisition of riboflavin, but merely an adaptation in the function of RNF.

In addition to the subunit acquisition and motif evolution, the adjustment in the midpoint potentials of the redox centers should have been one of the most important aspects necessary to complete the transition from RNF into Na^+^-NQR. The forward reaction catalyzed by RNF involves the electron transfer from Ferredoxin (*Em* = −500 mV) to NADH (*Em* = −320 mM) [62]. In Na^+^-NQR, the electrons move downhill from NADH to ubiquinone (*Em* = +90mV), following a pathway in which most redox centers have midpoint potentials higher than NADH [93], [94]. Hence, in order for the covalently -bound FMN molecules (and presumably for riboflavin too) to become functional in Na^+^-NQR, a significant change in the redox potential should have occurred, increasing the potentials probably by 50–490 mV, depending on the specific cofactor. In most proteins, the control of the redox potential of the centers is achieved by modifying the environment surrounding the cofactors. For instance, classic studies on Myoglobin have shown that the mutation of valine 68, which is close to the heme group, to aspartate or asparagine decreases the midpoint potential by 200 or 80 mV, respectively [95]. In flavin proteins, the dielectric constant of the immediate environment [96] and the interactions with aromatic residues have been reported to affect strongly the midpoint potential of the flavin cofactors [97], [98]. Thus, it is possible that in the evolution of Na^+^-NQR the environment of the flavins was modified, replacing negatively charged residues and decreasing the polarity of the flavin environment, which will increase the midpoint potential of the three flavin cofactors.

## Conclusion

The distribution and the phylogenetic history of the Na^+^-NQR subunits in different bacterial lineages suggest that the *nqr* operon, and subsequently the Na^+^-NQR complex, is the outcome of an ancestral duplication of the homologous *rnf* operon. Here, we present evidence that suggest a series of molecular events, comprising a specific partial gene loss, a gene fusion and enzymatic recruitment, that occurred during the divergence and neo-fuctionalization of the ancestral versatile RNF complex into the specialized sodium pump that became the modern Na^+^-NQR assemblage. Moreover, our phylogenic survey indicates that the putative duplication of the *rnf* operon occurred in the C/B group, and later the ancestral *nqr* operon dispersed via independent HGT events into different proteobacterial groups, and Chlamydiae and Planctomyces. This model is consistent with the idea that genes with related functions and organized in clusters (i.e., operons) are prone material for successful HGT, given that the encoded products, as an effective functional unit, are potentially able to confer novel metabolic traits, such as sodium pumping coupled to electron transport. Our analyses highlight the major relevance that duplication of genes, or clusters of genes, has in the emergence and consolidation of evolutionary innovations. In this case underlying the diversification and expansion of the bacterial metabolic capabilities under particular ecologic scenarios, such as the use of sodium gradients across membranes to generate electromotive forces when proton “inverted” (i.e., alkaline outside) gradients prevail in the surroundings. Our model posits that the emergence, and later independent recruitments, of the Na^+^-NQR allowed different types of bacteria with diverse metabolic adaptations to take advantage of the abundance of Na^+^ ions in particular habitats, such as marine, alkaline and intracellular environments. Finally, our work invites to thoroughly investigate the presence of paralogous operons in prokaryotic genomes to study the magnitude that duplication of gene clusters have had in the diversification and expansion of the microbial primary metabolism.

## Supporting Information

Figure S1 Alignment of NqrA and RnfC subunits. Secondary structure prediction of *Anaerophaga thermohalophila* (Bacteroidetes) RnfC and NqrA. Figure shows the secondary structure prediction consensus of eight algorithms, using the Network Protein Sequence Analysis software. The conserved residues of RnfC involved in NADH and FAD binding sites, as well as the cysteine residues involved in the 4Fe-4S centers attachement are highlighted in black.
(PDF)

Figure S2 Rooted phylogenetic tree of NqrB subunit. Maximum Likelihood phylogenetic analysis of the NqrB subunit of the Na^+^-NQR complex and the homolog RnfD subunit from the RNF complex. The tree was rooted using the RnfD clade as outgroup. Numbers near nodes indicate RaxML bootstrap branch support values (when ≥50%). Branch lengths are proportional to the number of substitutions per site. NCBI GI numbers precede the taxa identification.
(PDF)

Figure S3 Rooted phylogenetic tree of NqrF subunit. Maximum Likelihood phylogenetic analysis of the NqrF subunit of the Na^+^-NQR complex and sequences of the reductase subunit of aromatic monooxigenases (AMOr), which are the closes know homologues of NqrF. The tree was rooted using the AMOr (benC/ToL) proteins as outgroup. Numbers near nodes indicate RaxML bootstrap branch support values (when ≥50%). Branch lengths are proportional to the number of substitutions per site. NCBI GI numbers precede the taxa identification.
(PDF)

Figure S4 Rooted phylogenetic tree of NqrD subunit. Maximum Likelihood phylogenetic analysis of the NqrD subunit of the Na^+^-NQR complex and the homolog RnfE subunit from the RNF complex. The RnfE branch was defined as outgroup. Numbers near nodes indicate RaxML bootstrap branch support values (when ≥50%). Branch lengths are proportional to the number of substitutions per site. NCBI GI numbers precede the taxa identification.
(PDF)

Figure S5 Rooted phylogenetic tree of NqrE subunit. Maximum Likelihood phylogenetic analysis of the NqrE subunit of the Na^+^-NQR complex and the homolog RnfA subunit from the RNF complex. The tree was rooted using the RnfA branch (100% BS) as outgroup. Numbers near nodes indicate RaxML bootstrap branch support values (when ≥50%). Branch lengths are proportional to the number of substitutions per site. NCBI GI numbers precede the taxa identification.
(PDF)

Figure S6 Unrooted phylogenetic tree of NqrD subunit. Maximum Likelihood tree of the subunit D (NqrD) of the Na^+^-NQR complex. Numbers near nodes indicate RaxML bootstrap branch support values (when ≥50%). Branch lengths are proportional to the number of substitutions per site.
(PDF)

Figure S7 Unrooted phylogenetic tree of NqrE subunit. Maximum Likelihood tree of the subunit NqrE of the Na^+^-NQR complex. Numbers near nodes indicate RaxML bootstrap branch support values (when ≥50%). Branch lengths are proportional to the number of substitutions per site.
(PDF)

Figure S8 Unrooted phylogenetic tree of NqrA subunit. Maximum Likelihood tree of the subunit A (NqrA) of the Na^+^-NQR complex. Numbers near nodes indicate RaxML bootstrap branch support values (when ≥50%). Branch lengths are proportional to the number of substitutions per site.
(PDF)

Figure S9 Unrooted phylogenetic tree of NqrC subunit. Maximum Likelihood tree of the subunit NqrC of the Na^+^-NQR complex. Numbers near nodes indicate RaxML bootstrap branch support values (when ≥50%). Branch lengths are proportional to the number of substitutions per site.
(PDF)

Table S1 List of the analyzed bacterial taxa harboring Na^+^-NQR complex.
(PDF)

Table S2 Similarity profiles of the Na^+^-NQR subunits with the closest homologues in RNF and AMOr.
(PDF)
