# Supplementary figures and images for: Origin and Evolution of the Sodium -Pumping NADH: Ubiquinone Oxidoreductase
Source: PLoS One. 2014 May 8;9(5):e96696. doi: 10.1371/journal.pone.0096696 (PMC4014512; doi:10.1371/journal.pone.0096696)

### Figure S1

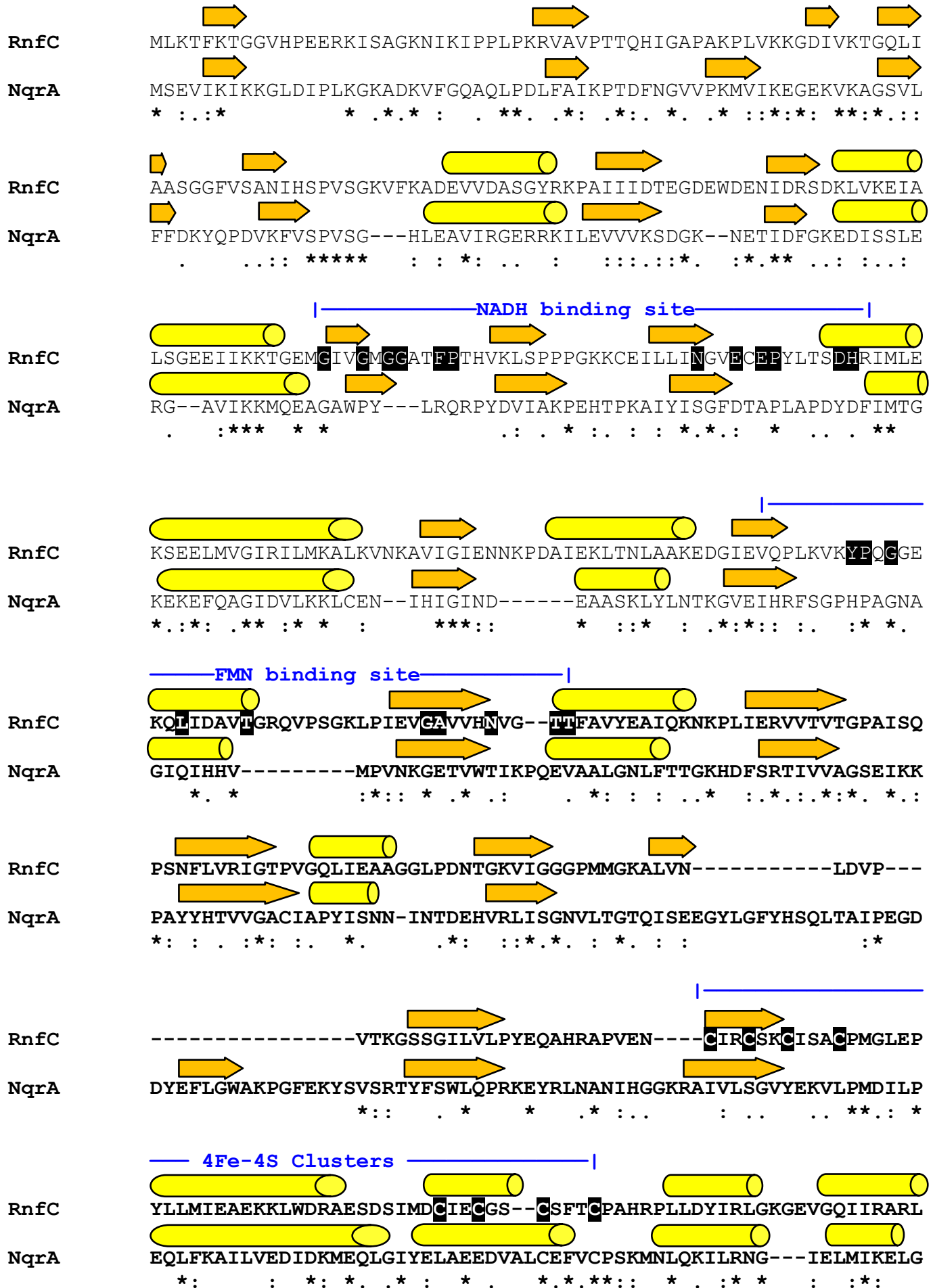

Supplement: Figure S1 — Alignment of NqrA and RnfC subunits. Secondary structure prediction of Anaerophaga thermohalophila (Bacteroidetes) RnfC and NqrA. Figure shows the secondary structure prediction consensus of eight algorithms, using the Network Protein Sequence Analysis software. The conserved residues of RnfC involved in NADH and FAD binding sites, as well as the cysteine residues involved in the 4Fe-4S centers attachement are highlighted in black. (PDF) [file pone.0096696.s001.pdf]

Figure S2

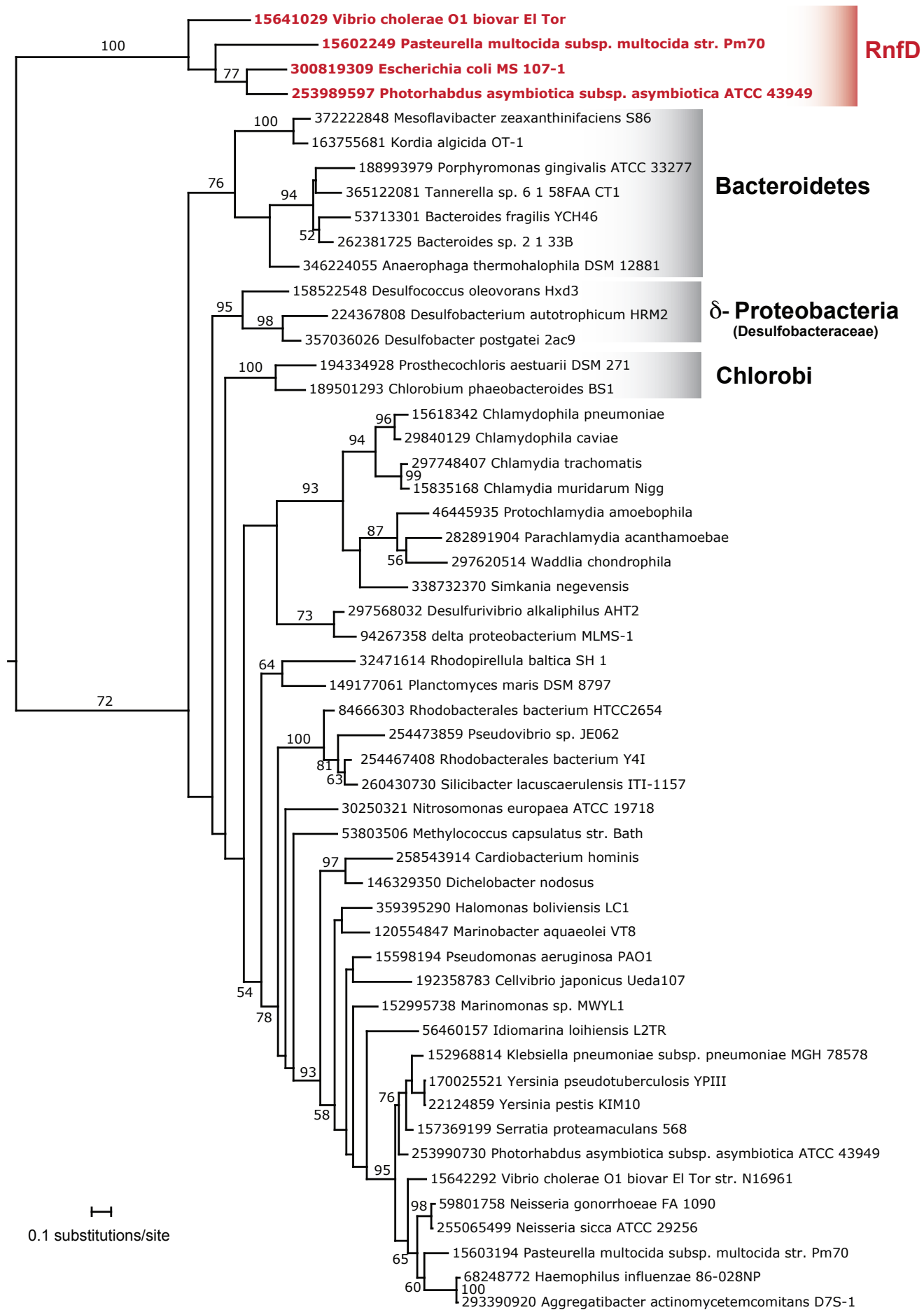

Supplement: Figure S2 — Rooted phylogenetic tree of NqrB subunit. Maximum Likelihood phylogenetic analysis of the NqrB subunit of the Na+-NQR complex and the homolog RnfD subunit from the RNF complex. The tree was rooted using the RnfD clade as outgroup. Numbers near nodes indicate RaxML bootstrap branch support values (when ≥50%). Branch lengths are proportional to the number of substitutions per site. NCBI GI numbers precede the taxa identification. (PDF) [file pone.0096696.s002.pdf]

**Figure S3**

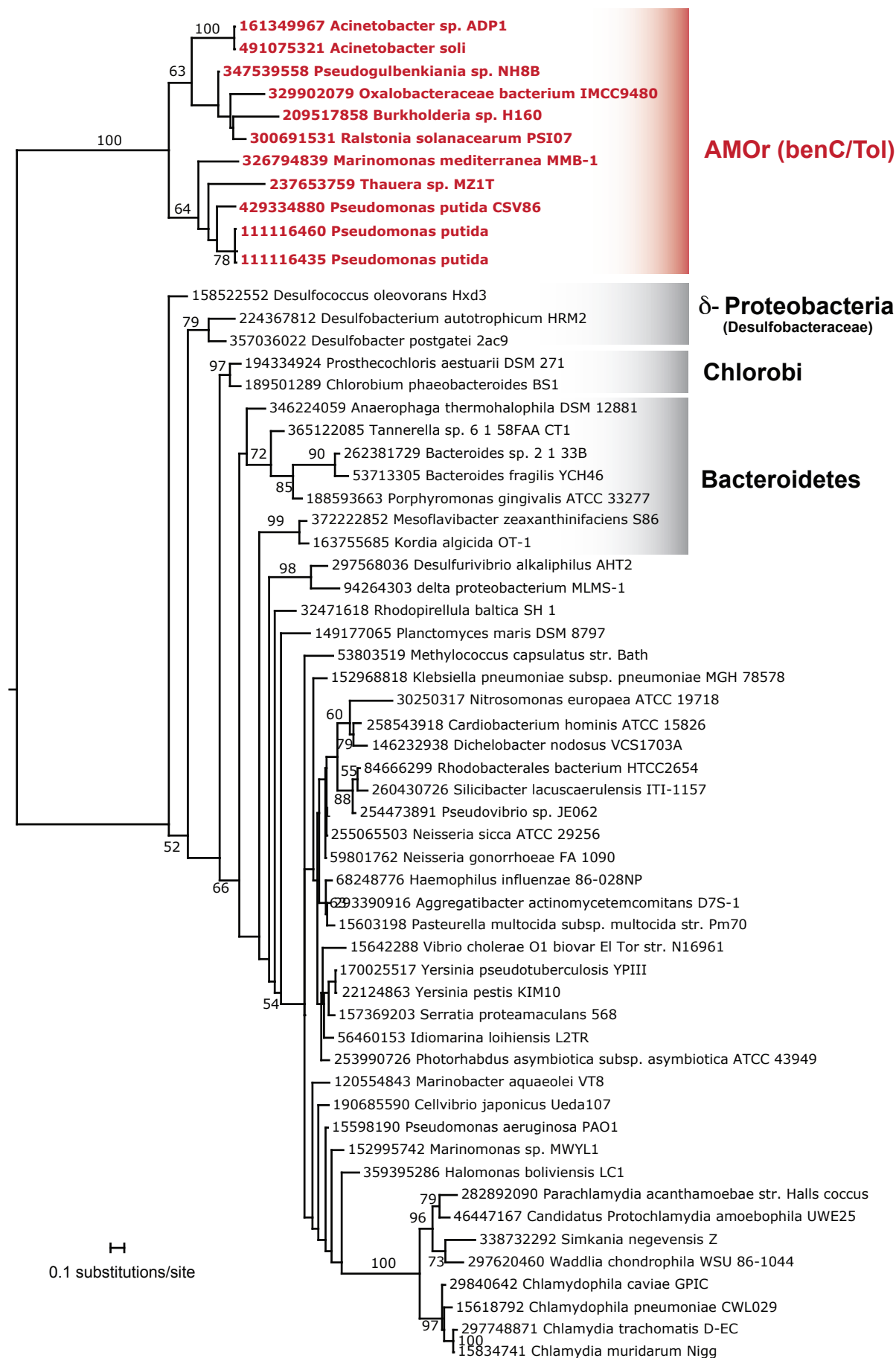

Supplement: Figure S3 — Rooted phylogenetic tree of NqrF subunit. Maximum Likelihood phylogenetic analysis of the NqrF subunit of the Na+-NQR complex and sequences of the reductase subunit of aromatic monooxigenases (AMOr), which are the closes know homologues of NqrF. The tree was rooted using the AMOr (benC/ToL) proteins as outgroup. Numbers near nodes indicate RaxML bootstrap branch support values (when ≥50%). Branch lengths are proportional to the number of substitutions per site. NCBI GI numbers precede the taxa identification. (PDF) [file pone.0096696.s003.pdf]

Figure S4

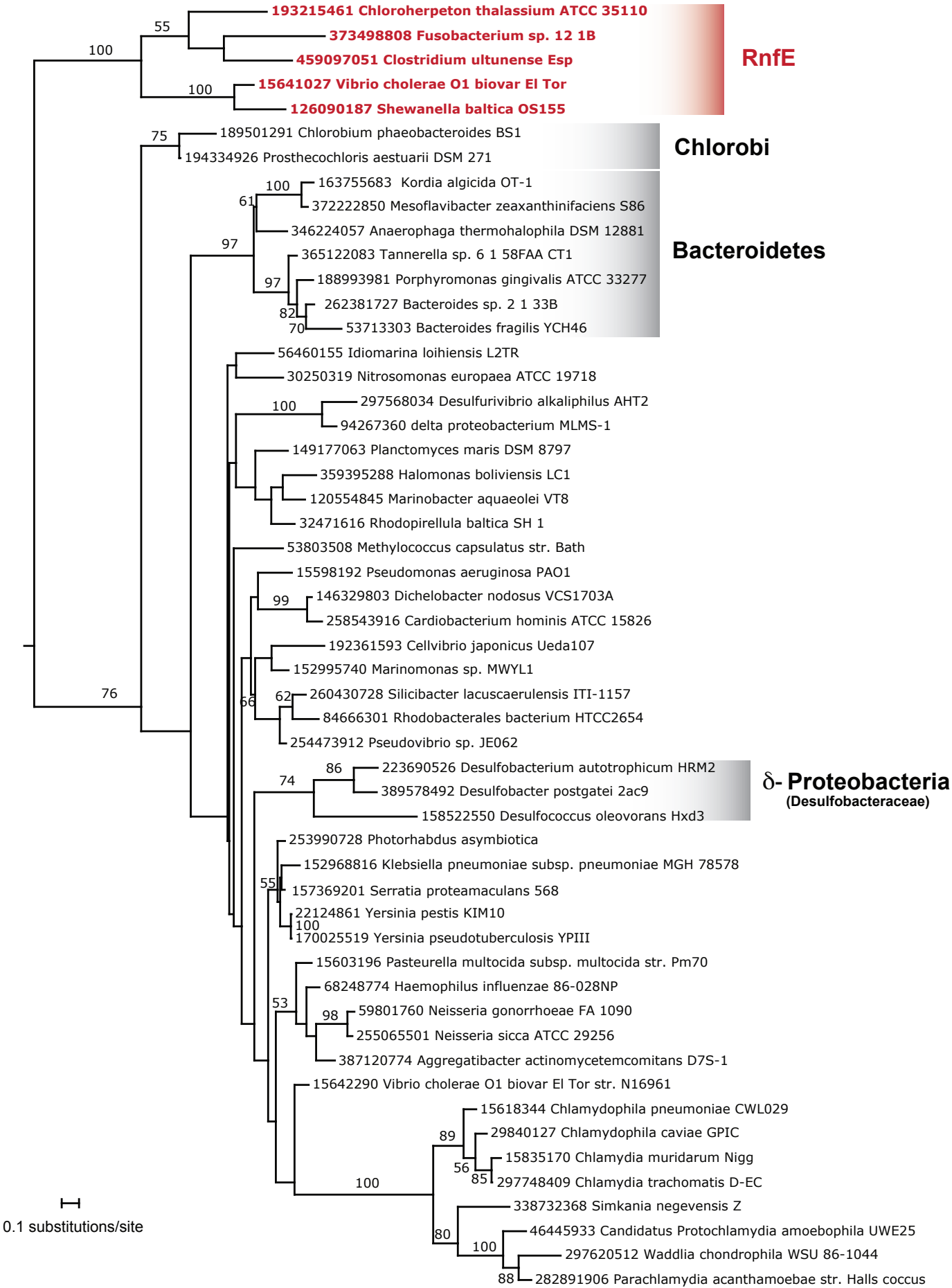

Supplement: Figure S4 — Rooted phylogenetic tree of NqrD subunit. Maximum Likelihood phylogenetic analysis of the NqrD subunit of the Na+-NQR complex and the homolog RnfE subunit from the RNF complex. The RnfE branch was defined as outgroup. Numbers near nodes indicate RaxML bootstrap branch support values (when ≥50%). Branch lengths are proportional to the number of substitutions per site. NCBI GI numbers precede the taxa identification. (PDF) [file pone.0096696.s004.pdf]

**Figure S5**

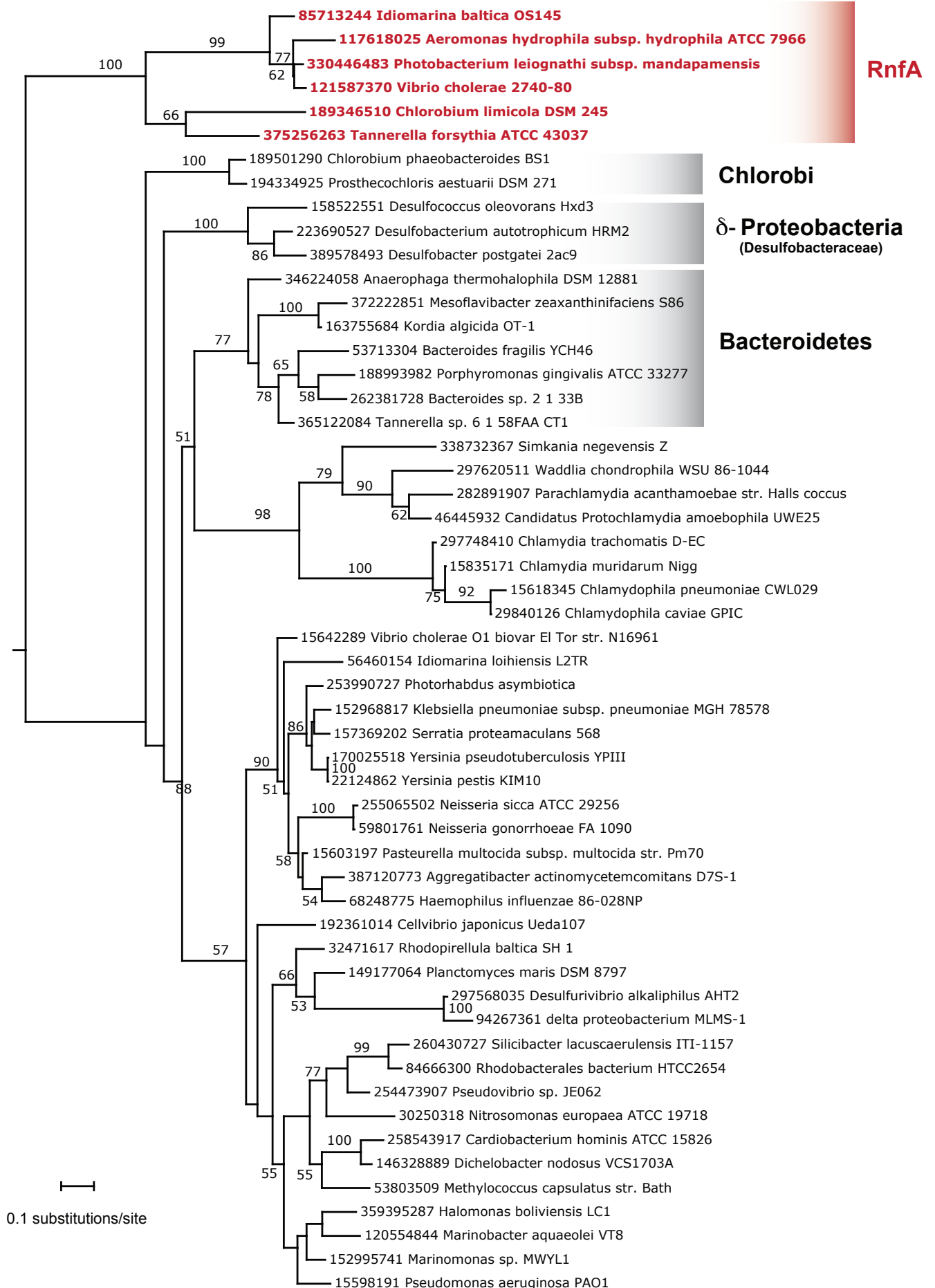

Supplement: Figure S5 — Rooted phylogenetic tree of NqrE subunit. Maximum Likelihood phylogenetic analysis of the NqrE subunit of the Na+-NQR complex and the homolog RnfA subunit from the RNF complex. The tree was rooted using the RnfA branch (100% BS) as outgroup. Numbers near nodes indicate RaxML bootstrap branch support values (when ≥50%). Branch lengths are proportional to the number of substitutions per site. NCBI GI numbers precede the taxa identification. (PDF) [file pone.0096696.s005.pdf]

**Figure S6**

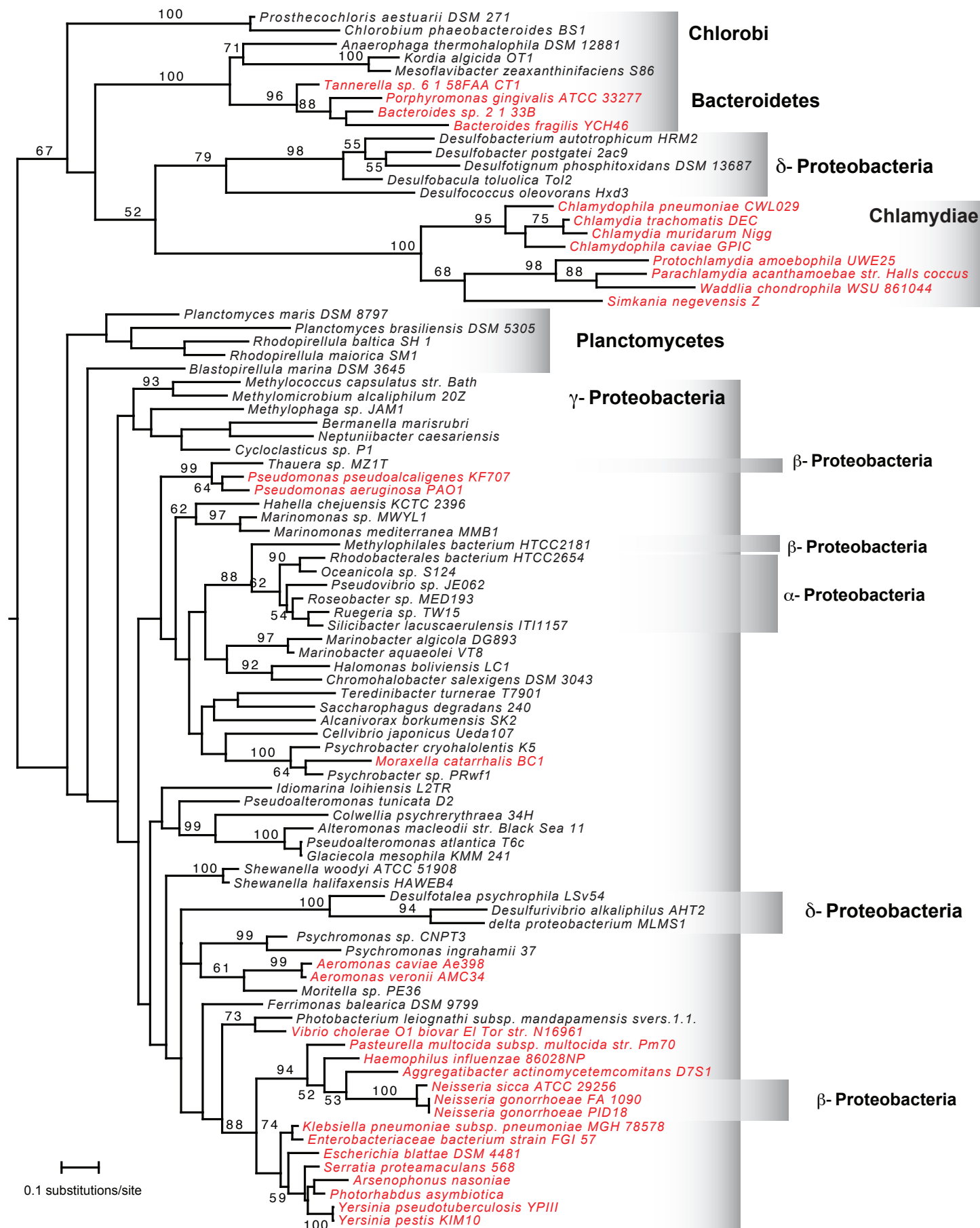

Supplement: Figure S6 — Unrooted phylogenetic tree of NqrD subunit. Maximum Likelihood tree of the subunit D (NqrD) of the Na+-NQR complex. Numbers near nodes indicate RaxML bootstrap branch support values (when ≥50%). Branch lengths are proportional to the number of substitutions per site. (PDF) [file pone.0096696.s006.pdf]

Figure S7

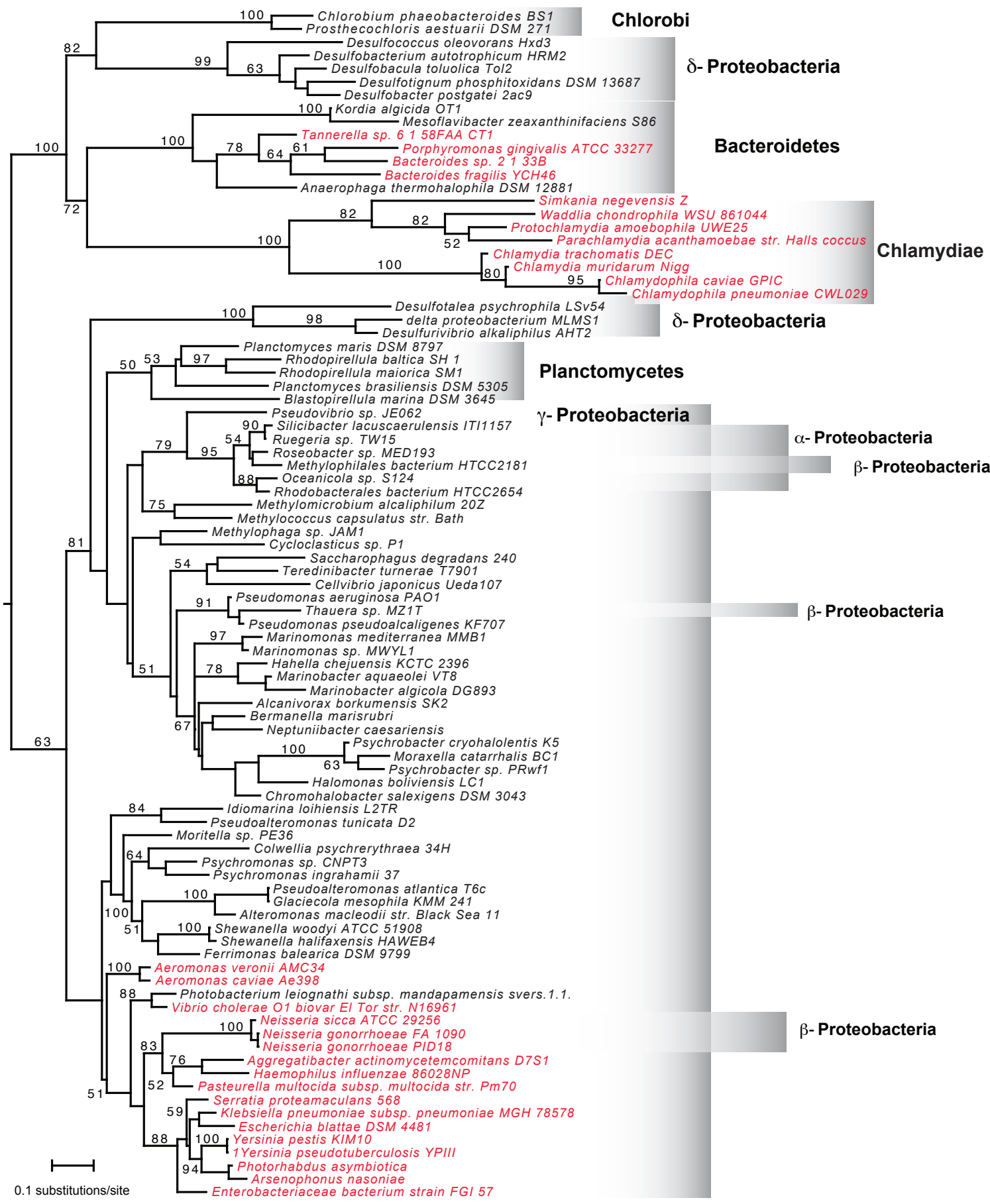

Supplement: Figure S7 — Unrooted phylogenetic tree of NqrE subunit. Maximum Likelihood tree of the subunit NqrE of the Na+-NQR complex. Numbers near nodes indicate RaxML bootstrap branch support values (when ≥50%). Branch lengths are proportional to the number of substitutions per site. (PDF) [file pone.0096696.s007.pdf]

Figure S8

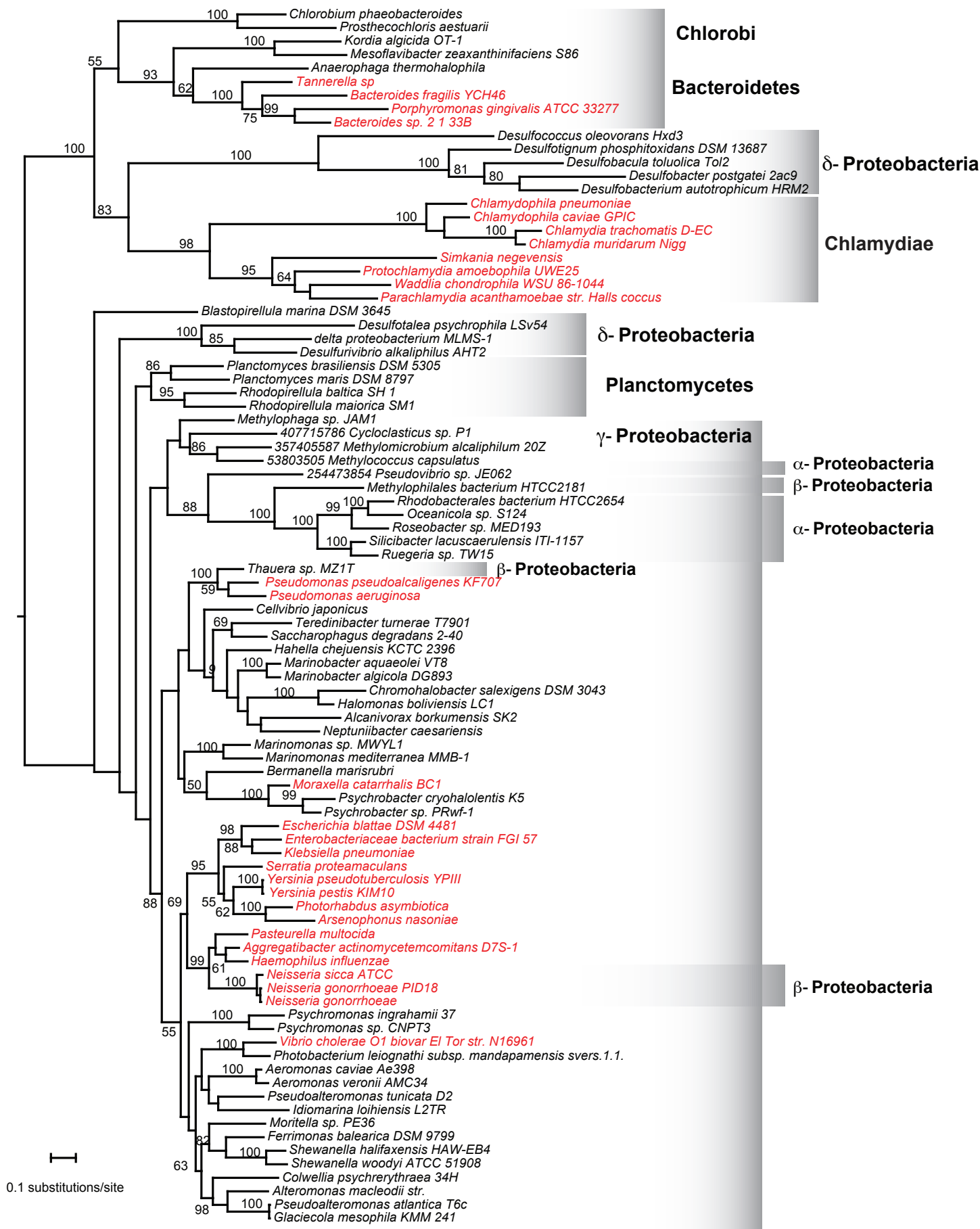

Supplement: Figure S8 — Unrooted phylogenetic tree of NqrA subunit. Maximum Likelihood tree of the subunit A (NqrA) of the Na+-NQR complex. Numbers near nodes indicate RaxML bootstrap branch support values (when ≥50%). Branch lengths are proportional to the number of substitutions per site. (PDF) [file pone.0096696.s008.pdf]

Figure S9

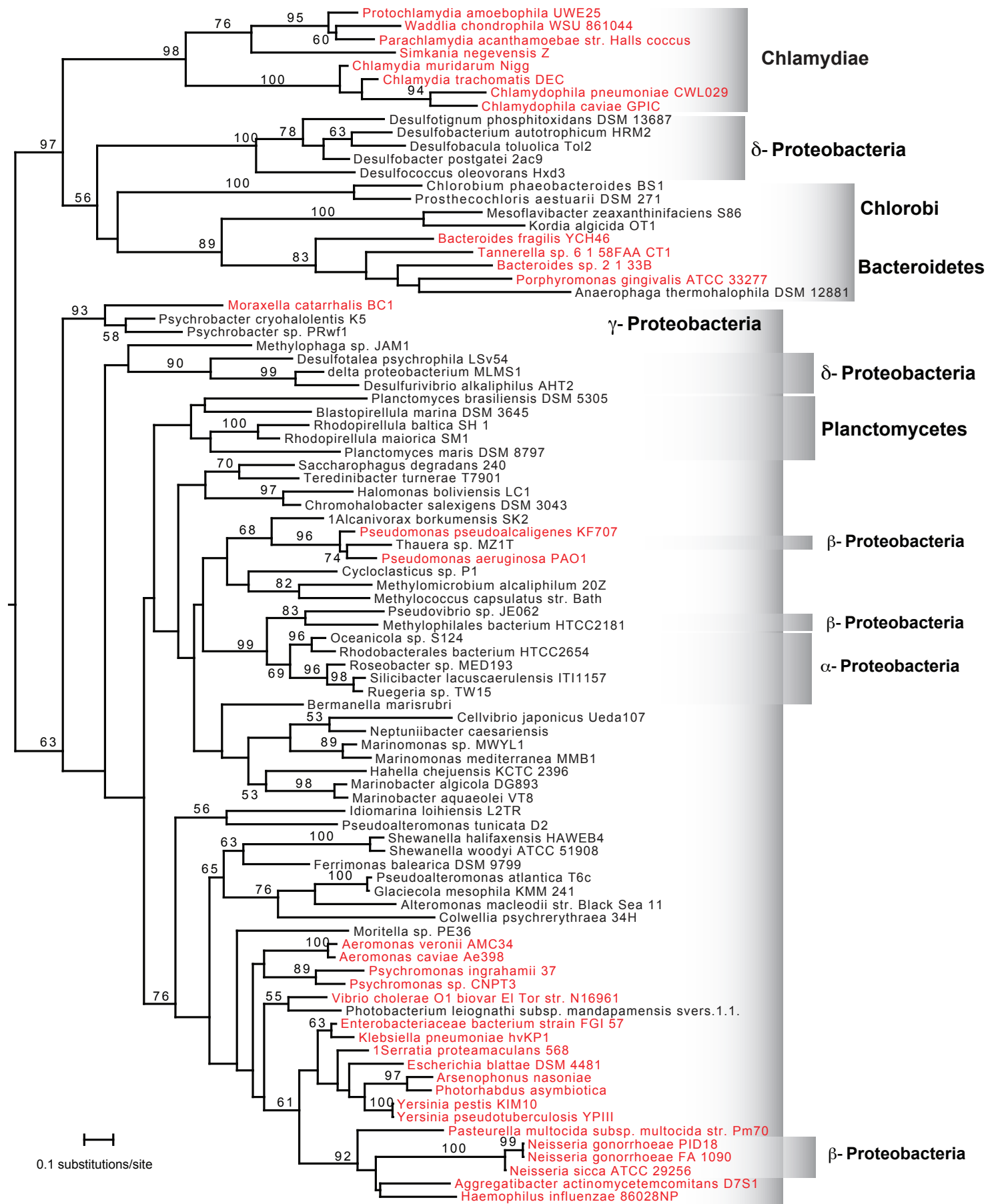

Supplement: Figure S9 — Unrooted phylogenetic tree of NqrC subunit. Maximum Likelihood tree of the subunit NqrC of the Na+-NQR complex. Numbers near nodes indicate RaxML bootstrap branch support values (when ≥50%). Branch lengths are proportional to the number of substitutions per site. (PDF) [file pone.0096696.s009.pdf]
