# Supplementary material for: Origin and Evolution of the Sodium -Pumping NADH: Ubiquinone Oxidoreductase
Source: PLoS One. 2014 May 8;9(5):e96696. doi: 10.1371/journal.pone.0096696 (PMC4014512; doi:10.1371/journal.pone.0096696)
Supplement: Table S1 — List of the analyzed bacterial taxa harboring Na+-NQR complex. (PDF) [file pone.0096696.s010.pdf]

Supplementary Table SI. List of the analyzed bacterial taxa harboring Na+-NQR complex.

Group	Species	Habitat	Na+-NQR subunits GI numbers (NCBI)					
			NqrA	NqrB	NqrF	NqrC	NqrD	NqrE
α−proteobacteria-Rhodobacterales	*Pseudovibrio sp. JE062*	FL M	254473854	254473859	254473891	254473895	254473912	254473907
α−proteobacteria-Rhodobacterales	*Maritimibacter alkaliphilus str. HTCC2654*	FL M	84666304	84666303	84666299	84666302	84666301	84666300
α−proteobacteria-Rhodobacterales	*Oceanicola sp. S124*	FL M	372278659	372278658	372278654	372278657	372278656	372278655
α−proteobacteria-Rhodobacterales	*Ruegeria sp. TW15*	FL M	346993341	346993342	346993346	346993343	346993344	346993345
α−proteobacteria-Rhodobacterales	*Roseobacter sp. MED193*	FL M	86138122	86138121	86138117	86138120	86138119	86138118
α−proteobacteria-Rhodobacterales	*Silicibacter lacuscaerulensis ITI-1157*	FL non-M Halo	260430731	260430730	260430726	260430729	260430728	260430727
β−proteobacteria-Methylophilales	*Methylophilales bacterium HTCC2181*	FL M	118595098	118595097	118595093	118595096	118595095	118595094
β−proteobacteria-Rhodocyclales	*Thauera sp. MZ1T*	FL non-M	217969242	217969243	217969247	217969244	217969245	217969246
β−proteobacteria-Neisseriales	*Neisseria gonorrhoeae PID18*	H Path	268601858	268601859	268601863	268601860	268601861	268601862
β−proteobacteria-Neisseriales	*Neisseria sicca ATCC 29256*	H mic	255065498	255065499	255065503	255065500	255065501	255065502
β−proteobacteria-Neisseriales	*Neisseria gonorrhoeae FA 1090*	H Path	59801757	59801758	59801762	59801759	59801760	59801761
δ−proteobacteria-Desulfobacterales-Desulfobacteraceae	*Desulfobacter postgatei 2ac9*	FL M	357036027	357036026	357036022	389578491	389578492	389578493
δ−proteobacteria-Desulfobacterales-Desulfobacteraceae	*Desulfobacterium autotrophicum HRM2*	FL M	224367807	224367808	224367812	223690525	223690526	223690527
δ−proteobacteria-Desulfobacterales-Desulfobacteraceae	*Desulfobacula toluolica Tol2*	FL M	408420322	408420321	408420317	408420320	408420319	408420318
δ−proteobacteria-Desulfobacterales-Desulfobacteraceae	*Desulfotignum phosphitoxidans DSM 13687*	FL M	476533602	476533603	476533607	476533604	476533605	476533606
δ−proteobacteria-Desulfobacterales-Desulfobacteraceae	*Desulfococcus oleovorans Hxd3*	FL non-M Halo	158522547	158522548	158522552	158522549	158522550	158522551
δ−proteobacteria-Desulfobacterales-Desulfobulbaceae	*Desulfotalea psychrophila LSv54*	FL M	51245654	51245655	51245659	51245656	51245657	51245658
δ−proteobacteria-Desulfobacterales-Desulfobulbaceae	*Desulfurivibrio alkaliphilus AHT2*	FL non-M Halo	297568031	297568032	297568036	297568033	297568034	297568035
δ−proteobacteria-unclassified	*delta proteobacterium MLMS-1*	FL non-M	94267357	94267358	94264303	94267359	94267360	94267361
γ−proteobacteria-Aeromonadales	*Aeromonas caviae Ae398*	H Path	334703564	334703565	334703569	334703566	334703567	334703568
γ−proteobacteria-Aeromonadales	*Aeromonas veronii AMC34*	FL non-M, but H/A Path	423205572	423205571	423205567	423205570	423205569	423205568
γ−proteobacteria-Alteromonadales	*Alteromonas macleodii str. 'Black Sea 11*	FL M	407701426	407701425	407701421	407701424	407701423	407701422
γ−proteobacteria-Alteromonadales	*Marinobacter aquaeolei VT8*	FL M	120554848	120554847	120554843	120554846	120554845	120554844
γ−proteobacteria-Alteromonadales	*Colwellia psychrerythraea 34H*	FL M	71279370	71279883	71281144	71278488	71282119	71282541
γ−proteobacteria-Alteromonadales	*Ferrimonas balearica DSM 9799*	FL M	308048613	308048614	308048618	308048615	308048616	308048617
γ−proteobacteria-Alteromonadales	*Glaciecola mesophila KMM 241*	M	410626984	410626985	410626989	410626986	410626987	410626988
γ−proteobacteria-Alteromonadales	*Idiomarina loihiensis L2TR*	FL M Halo	56460158	56460157	56460153	56460156	56460155	56460154
γ−proteobacteria-Alteromonadales	*Marinobacter algicola DG893*	IntCel/Protist	149376581	149376580	149376576	149376579	149376578	149376577
γ−proteobacteria-Alteromonadales	*Moritella sp. PE36*	FL M	149911095	149911094	149911090	149911093	149911092	149911091
γ−proteobacteria-Alteromonadales	*Pseudoalteromonas atlantica T6c*	FL M	109896781	109896782	109896786	109896783	109896784	109896785
γ−proteobacteria-Alteromonadales	*Pseudoalteromonas tunicata D2*	FL M	88858245	88858244	88858240	88858243	88858242	88858241
γ−proteobacteria-Alteromonadales	*Psychromonas ingrahamii 37*	FL M	119944517	119944518	119944522	119944519	119944520	119944521
γ−proteobacteria-Alteromonadales	*Psychromonas sp. CNPT3*	FL M	470479877	470479876	470479872	470479875	470479874	470479873
γ−proteobacteria-Alteromonadales	*Saccharophagus degradans 2-40*	FL M	90021442	90021443	90021447	90021444	90021445	90021446
γ−proteobacteria-Alteromonadales	*Shewanella halifaxensis HAW-EB4*	FL M	167625099	167625098	167625094	167625097	167625096	167625095
γ−proteobacteria-Alteromonadales	*Shewanella woodyi ATCC 51908*	M	170727950	170727949	170727945	170727948	170727947	170727946
γ−proteobacteria-Alteromonadales	*Teredinibacter turnerae T7901*	M	254786006	254786007	254786011	254786008	254786009	254786010
γ−proteobacteria-Enterobacteriales	*Arsenophonus nasoniae*	A Path	284007777	284007776	284007772	284007775	284007774	284007773
γ−proteobacteria-Enterobacteriales	*Enterobacteriaceae bacterium strain FGI 57*	FL non-M	440288960	440288959	440288955	440288958	440288957	440288956
γ−proteobacteria-Enterobacteriales	*Escherichia blattae DSM 4481*	A Path	387890413	387890412	387890408	387890411	387890410	387890409
γ−proteobacteria-Enterobacteriales	*Klebsiella pneumoniae subsp. pneumoniae MGH 78578*	H/A Path	152968813	152968814	152968818	152968815	152968816	152968817
γ−proteobacteria-Enterobacteriales	*Photorhabdus asymbiotica*	H Path	253990731	253990730	253990726	253990729	253990728	253990727
γ−proteobacteria-Enterobacteriales	*Serratia proteamaculans 568*	IntCell/Protist	157369198	157369199	157369203	157369200	157369201	157369202
γ−proteobacteria-Enterobacteriales	*Yersinia pestis KIM10+*	H Path	22124858	22124859	22124863	22124860	22124861	22124862
γ−proteobacteria-Enterobacteriales	*Yersinia pseudotuberculosis YPIII*	H Path	170025522	170025521	170025517	170025520	170025519	170025518
γ−proteobacteria-Methylococcales	*Methylomicrobium alcaliphilum 20Z*	FL non-M Halo	357405587	357405586	357405582	357405585	357405584	357405583
γ−proteobacteria-Methylococcales	*Methylococcus capsulatus str. Bath*	FL non-M	53803505	53803506	53803519	53803507	53803508	53803509
γ−proteobacteria-Oceanospirillales	*Alcanivorax borkumensis SK2*	FL M	110833893	110833894	110833898	110833895	110833896	110833897
γ−proteobacteria-Oceanospirillales	*Bermanella marisrubri*	FL M	94499514	94499515	94499519	94499516	94499517	94499518
γ−proteobacteria-Oceanospirillales	*Chromohalobacter salexigens DSM 3043*	FL M Halo	92113693	92113694	92113698	92113695	92113696	92113697
γ−proteobacteria-Oceanospirillales	*Hahella chejuensis KCTC 2396*	FL M	83645463	83645464	83645468	83645465	83645466	83645467
γ−proteobacteria-Oceanospirillales	*Halomonas boliviensis LC1*	FL non-M Halo	359395291	359395290	359395286	359395289	359395288	359395287
γ−proteobacteria-Oceanospirillales	*Marinomonas mediterranea MMB-1*	FL M	326546602	326546601	326546597	326546600	326546599	326546598
γ−proteobacteria-Oceanospirillales	*Marinomonas sp. MWYL1*	FL M Halo	152995737	152995738	152995742	152995739	152995740	152995741
γ−proteobacteria-Oceanospirillales	*Neptuniibacter caesariensis*	FL M	89094302	89094303	89094307	89094304	89094305	89094306
γ−proteobacteria-Pasteurellales	*Aggregatibacter actinomycetemcomitans D7S-1*	H/A Path	293390921	293390920	293390916	387120775	387120774	387120773
γ−proteobacteria-Pasteurellales	*Haemophilus influenzae 86-028NP*	H Path	68248771	16272132	68248776	68248773	68248774	68248775
γ−proteobacteria-Pasteurellales	*Pasteurella multocida subsp. multocida str. Pm70*	H Path	15603193	15603194	15603198	15603195	15603196	15603197
γ−proteobacteria-Pseudomonadales	*Cellvibrio japonicus Ueda107*	FL non-M	192359833	192358783	190685590	192362189	192361593	192361014
γ−proteobacteria-Pseudomonadales	*Moraxella catarrhalis BC1*	H Path	416237647	416237648	416237652	416237649	416237650	416237651
γ−proteobacteria-Pseudomonadales	*Pseudomonas aeruginosa PAO1*	H Path	15598195	15598194	15598190	15598193	15598192	15598191
γ−proteobacteria-Pseudomonadales	*Pseudomonas pseudoalcaligenes KF707*	FL non-M	443472679	443472678	443472674	443472677	443472676	443472675
γ−proteobacteria-Pseudomonadales	*Psychrobacter cryohalolentis K5*	FL M	93007248	93007249	93007253	93007250	93007251	93007252
γ−proteobacteria-Pseudomonadales	*Psychrobacter sp. PRwf-1*	M	148651917	148651916	148651912	148651915	148651914	148651913
γ−proteobacteria-Thiotrichales	*Cycloclasticus sp. P1*	FL M	407715786	407715787	407715791	407715788	407715789	407715790
γ−proteobacteria-Thiotrichales	*Methylophaga sp. JAM1*	FL M	387128366	387128367	387128371	387128368	387128369	387128370
γ−proteobacteria-Vibrionales	*Photobacterium leiognathi subsp. mandapamensis svers.1.1*	FL M	330445290	330445291	330445295	330445292	330445293	330445294
γ−proteobacteria-Vibrionales	*Vibrio cholerae O1 biovar El Tor str. N16961*	M/H Path	15642293	15642292	15642288	15642291	15642290	15642289
Bacteroidetes/Chlorobi-Bacteroidetes	*Kordia algicida OT-1'*	FL M	163755680	163755681	163755685	163755682	163755683	163755684
Bacteroidetes/Chlorobi-Bacteroidetes	*Mesoflavibacter zeaxanthinifaciens S86*	FL M	372222847	372222848	372222852	372222849	372222850	372222851
Bacteroidetes/Chlorobi-Bacteroidetes	*Anaerophaga thermohalophila DSM 12881*	FL non-M	346224054	346224055	346224059	346224056	346224057	346224058
Bacteroidetes/Chlorobi-Bacteroidetes	*Bacteroides sp. 2_1_33B*	H mic	262381724	262381725	262381729	262381726	262381727	262381728
Bacteroidetes/Chlorobi-Bacteroidetes	*Bacteroides fragilis YCH46*	H/A Path	60681549	53713301	53713305	53713302	53713303	53713304
Bacteroidetes/Chlorobi-Bacteroidetes	*Tannerella sp. 6_1_58FAA_CT1*	H Path	365122080	365122081	365122085	365122082	365122083	365122084
Bacteroidetes/Chlorobi-Bacteroidetes	*Porphyromonas gingivalis ATCC 33277*	H Path	188993978	188993979	188993983	188993980	188993981	188993982
Bacteroidetes/Chlorobi-Chlorobi	*Chlorobium phaeobacteroides BS1*	FL non-M	189501294	189501293	189501289	189501292	189501291	189501290
Bacteroidetes/Chlorobi-Chlorobi	*Prosthecochloris aestuarii DSM 271*	FL M	194334929	194334928	194334924	194334927	194334926	194334925
Chlamydiae-Chlamydiia-Chlamydiales	*Chlamydophila caviae GPIC*	A Path	29839771	29840129	29840642	29840128	29840127	29840126
Chlamydiae-Chlamydiia-Chlamydiales	*Waddlia chondrophila WSU 86-1044*	A Path	297622124	297620514	297620460	297620513	297620512	297620511
Chlamydiae-Chlamydiia-Chlamydiales	*Candidatus Protochlamydia amoebophila UWE25*	H Path	46445729	46445935	46447167	46445934	46445933	46445932
Chlamydiae-Chlamydiia-Chlamydiales	*Chlamydia trachomatis D-EC*	H Path	297748764	297748407	297748871	297748408	297748409	297748410
Chlamydiae-Chlamydiia-Chlamydiales	*Chlamydophila pneumoniae CWL029*	H Path	15618653	15618342	15618792	15618343	15618344	15618345
Chlamydiae-Chlamydiia-Chlamydiales	*Simkania negevensis Z*	H Path	336483587	338732370	338732292	338732369	338732368	338732367
Chlamydiae-Chlamydiia-Chlamydiales	*Chlamydia muridarum Nigg'*	H/A Path	15834627	15835168	15834741	15835169	15835170	15835171
Chlamydiae-Chlamydiia-Chlamydiales	*Parachlamydia acanthamoebae str. Hall's coccus*	IntCell/Protist	282890124	282891904	282892090	282891905	282891906	282891907
Planctomycetes-Planctomycetia	*Planctomyces maris DSM 8797*	FL M	149177060	149177061	149177065	149177062	149177063	149177064
Planctomycetes-Planctomycetia	*Rhodopirellula baltica SH 1*	FL M	32471613	32471614	32471618	32471615	32471616	32471617
Planctomycetes-Planctomycetia	*Rhodopirellula maiorica SM1*	FL M	470097590	470097589	470097585	470097588	470097587	470097586
Planctomycetes-Planctomycetia	*Blastopirellula marina DSM 3645*	FL M	87311095	87311096	87311100	87311097	87311098	87311099
Planctomycetes-Planctomycetia	*Planctomyces brasiliensis DSM 5305*	FL M Halo	325108117	325108118	325108122	325108119	325108120	325108121

A Path: Animal pathogen
FL M: Free-living marine
FL M Halo: Free-living marine, halophile
FL non-M: Free-living non-marine
FL non-M Halo: Free-living non-marine, halophile
FL non-M, but H/A Path: Free-living non-marine, opportunistic Human/Animal pathogen
H mic: Human microbiota
H Path: Human pathogen
H/A Path: Human/Animal pathogen
IntCell/Protist: Intracellular/protist-associated
M: Marine, commensal
M/H Path: Marine, opportunistic human pathogen
