# Supplementary material for: Origin and Evolution of the Sodium -Pumping NADH: Ubiquinone Oxidoreductase
Source: PLoS One. 2014 May 8;9(5):e96696. doi: 10.1371/journal.pone.0096696 (PMC4014512; doi:10.1371/journal.pone.0096696)
Supplement: Table S2 — Similarity profiles of the Na+-NQR subunits with the closest homologues in RNF and AMOr. (PDF) [file pone.0096696.s011.pdf]

**Table S2.** Similarity profiles of the $Na^{+}$-NQR subunits with the closest homologues in RNF and AMOr.

| | Homolog pairs | | | Gaps/Al | | PID 1 (%) | | PID 2 (%) | | PID 3 (%) | | PID 4 (%) | | AM PID ± STDV | |
|---|---|---|---|---|---|---|---|---|---|---|---|---|---|---|---|
| *Chlorobium phaeobacteroides* | *rnf* L | *nqr* L | AM L | B62 | B40 | B62 | B40 | B62 | B40 | B62 | B40 | B62 | B40 | B62 | B40 |
| *rnfC* *nqrA* | 441 | 451 | 446 | 142/517 | 198/545 | 18.8 | 19.1 | 25.9 | 30.0 | 22.0 | 23.6 | 21.7 | 23.3 | **22.1 ± 2.9** | **24.0 ± 4.5** |
| *rnfE* *nqrD* | 201 | 209 | 205 | 24/217 | 24/217 | 36.4 | 36.9 | 40.9 | 41.5 | 39.3 | 39.8 | 38.5 | 39.0 | 38.8 ± 1.9 | 39.3 ± 1.9 |
| *rnfA* *nqrE* | 195 | 208 | 201.5 | 13/208 | 13/208 | 39.9 | 40.4 | 42.6 | 43.1 | 42.6 | 43.1 | 41.2 | 41.7 | 41.6 ± 1.3 | 42.1 ± 1.3 |
| *rnfD* *nqrB* | 331 | 384 | 357.5 | 75/395 | 91/403 | 30.4 | 30.3 | 37.5 | 39.1 | 36.3 | 36.9 | 33.6 | 34.1 | 34.4 ± 3.1 | 35.1 ± 3.8 |
| *rnfG* *nqrC* | 181 | 308 | 244.5 | 73/231 | 65/227 | 19.5 | 18.5 | 28.5 | 25.9 | 24.9 | 23.2 | 23.1 | 17.2 | **24.0 ± 3.7** | **21.2 ± 4.1** |
| *pMo*[A] *nqrF* | 354 | 409 | 381.5 | 117/440 | 145/454 | 24.3 | 26.2 | 33.1 | 38.5 | 30.2 | 33.6 | 28.0 | 31.2 | **28.9 ± 3.7** | 32.4 ± 5.1 |
| *Anaerophaga thermohalophila* | | | | | | | | | | | | | | | |
| *rnfC* *nqrA* | 442 | 449 | 445.5 | 173/532 | 189/540 | 18.8 | 22.0 | 27.9 | 33.9 | 22.6 | 26.9 | 22.4 | 26.7 | **22.9 ± 3.7** | **27.4 ± 4.9** |
| *rnfE* *nqrD* | 194 | 220 | 207 | 32/223 | 32/223 | 31.8 | 31.8 | 37.2 | 37.2 | 36.6 | 36.6 | 34.3 | 34.3 | 35.0 ± 2.4 | 35.0 ± 2.4 |
| *rnfA* *nqrE* | 190 | 205 | 197.5 | 15/205 | 16/205 | 38.0 | 38.0 | 41.1 | 41.3 | 41.1 | 41.1 | 39.5 | 39.5 | 39.9 ± 1.5 | 40.0 ± 1.5 |
| *rnfD* *nqrB* | 339 | 386 | 362.5 | 89/407 | 98/411 | 27.8 | 28.5 | 35.5 | 37.4 | 33.3 | 34.5 | 31.2 | 32.3 | 32.0 ± 3.3 | 33.2 ± 3.7 |
| *rnfG* *nqrC* | 238 | 234 | 236 | 94/283 | 108/290 | 19.4 | 21.4 | 29.1 | 34.1 | 23.5 | 26.5 | 23.3 | 26.3 | **23.8 ± 4.0** | **27.1 ± 5.2** |
| *pMo*[A] *nqrF* | 353 | 419 | 386 | 132/452 | 148/460 | 24.6 | 27.0 | 34.7 | 39.7 | 31.4 | 35.1 | 28.8 | 32.1 | **29.9 ± 4.3** | 33.5 ± 5.3 |

*A pMO* from *Cupriavidus metallidurans CH34*

Protein sequence identity (number of identical residues) between *rnf* and *nqr* putative protein homolog pairs from *Chlorobium phaeobacteroides BS1* and *Anaerophaga thermohalophila*, respectively, was estimated with the global alignment software *Needle* (Needleman-Wunsch algorithm; 1) of *EMBOSS* [2] and considering alternatively the BLOSUM40 (B40) and BLOSUM62 (B62) substitution matrices [3]. Since aligned proteins sequences were not of the same length (*rnf* L versus *nqr* L), we used different strategies to correct sequence identity values [4]. We considered 1) the total number of aligned positions (Al) and gaps (PID 1); 2) the number of aligned identities and no identities excluding gaps (PID 2); 3) the length of the shorter aligned sequence (PID 3) and 4) the arithmetic mean (AM L) of the compared sequences length (PID 4) as alternative denominators to normalize the protein sequence identity [4].

## References


1. Needleman, S.B. and Wunsch, C.D. 1970. A general method applicable to the search for similarities in the amino acid sequence of two proteins. *Journal of Molecular Biology* 48: 443-453.
2. Rice, P., Longden, I. and Bleasby, A. 2000. EMBOSS: The European Molecular Biology Open Software Suite. *Trends in Genetics* 16: 276-277
3. Henikoff, S. and Henikoff, J.G. 1992. Amino acid substitution matrices from protein blocks. *Proc Natl Acad Sci U S A*. 89:10915-10919.
4. May, A.C. 2004. Percent sequence identity: the need to be explicit. *Structure*. 12: 737-738.
